# Alterations in gene expression in T1α null lung: a model of deficient alveolar sac development

**DOI:** 10.1186/1471-213X-6-35

**Published:** 2006-07-25

**Authors:** Guetchyn Millien, Avrum Spira, Anne Hinds, Junling Wang, Mary C Williams, Maria I Ramirez

**Affiliations:** 1 Pulmonary Center, Department of Medicine, Boston University School of Medicine, Boston, MA, USA; 2 Bioinformatics Program, Boston University College of Engineering, Boston, MA, USA; 3 Department of Pathology and Laboratory Medicine, Boston University School of Medicine, Boston, MA, USA; 4 Department of Anatomy, Boston University School of Medicine, Boston, MA, USA

## Abstract

**Background:**

Development of lung alveolar sacs of normal structure and size at late gestation is necessary for the gas exchange process that sustains respiration at birth. Mice lacking the lung differentiation gene T1α [T1α(-/-)] fail to form expanded alveolar sacs, resulting in respiratory failure at birth. Since little is known about the molecular pathways driving alveolar sacculation, we used expression microarrays to identify genes altered in the abnormal lungs and, by inference, may play roles in normal lung morphogenesis.

**Results:**

Altered expression of genes related to cell-cell interaction, such as ephrinA3, are observed in T1α(-/-) at E18.5. At term, FosB, Egr1, MPK-1 and Nur77, which can function as negative regulators of the cell-cycle, are down-regulated. This is consistent with the hyperproliferation of peripheral lung cells in term T1α (-/-) lungs reported earlier. Biochemical assays show that neither PCNA nor p21 are altered at E18.5. At term in contrast, PCNA is increased, and p21 is decreased.

**Conclusion:**

This global analysis has identified a number of candidate genes that are significantly altered in lungs in which sacculation is abnormal. Many genes identified were not previously associated with lung development and may participate in formation of alveolar sacs prenatally.

## Background

Lung development starts in mice at embryonic day 9.5 (E9.5). By E16.5, airways have extensively grown and branched to form the bronchial tree. Between E16.5 and term (E20.5) lung cell proliferation is gradually reduced, and the distal lung undergoes significant morphogenetic changes to form the alveolar sacs. While a population of distal epithelial cells flattens, thins, and spreads to form type I cells, other distal epithelial cells remain cuboidal, acquire surfactant filled lamellar bodies and differentiate into type II cells. Differentiation of epithelial cells is accompanied by vascular remodeling and thinning of the mesenchyme, and results in enlargement of the diameter and surface area of the alveolar sacs. Overall this process is known as sacculation, and it is critical to increase the efficiency of fluid absorption and gas exchange processes at birth [1-3].

Very little is known about the molecular regulation of sacculation in normal animals. Abnormal sacculation has been reported in many genetically altered animals carrying null mutations, or transgenes that mis- or over-express growth factors, transcription factors, and other regulatory molecules. These molecular abnormalities result in formation of alveolar spaces that are either too small, as in glucocorticoid receptor (GR) [4], corticotropin releasing hormone (CRH) [5], and Sp3 knockout mice [6], and double p21(+/-)p57(+/-) and p21(-/-)p57(+/-) mice [7], or too large as in gp330 knockout mouse [8], the SP-C promoter-Bmp4 mouse [9], and SP-C promoter-GATA6 mouse [10]. It is interesting that both extremes of alveolar sac size can result in death of the newborn shortly after birth due to respiratory failure. Collectively these observations suggest that formation of alveolar sacs of appropriate dimensions, surface area, and thickness is of fundamental importance in lung organogenesis and is critical for survival.

We have previously shown that mice carrying a null mutation of the T1α gene fail to form expanded alveolar sacs near term and die at birth due to an inability to inflate their lungs with the first few breaths [11]. In normal late fetal and adult lungs, T1α protein is uniquely expressed in the apical membrane of type I alveolar epithelial cells, which form over 90% of the alveolar surface that is specialized for gas exchange [12-14]. In the absence of this protein the alveolar sacs still form but they are narrower than normal and do not properly expand at birth. This abnormality appears to be linked to deficient differentiation of type I cells. This was indicated by the presence of fewer attenuated type I cells and reduced expression of Aqp-5, another type I cell marker gene. Secreted surfactant and surfactant gene expression patterns indicate normal differentiation of type II cells in T1α (-/-) lungs.

Some insights into the process of alveolar sacculation in normal animals come from gene expression microarray data using lungs of normal mice at different developmental time points from embryonic day 9 through postnatal week 4 [15]. This study shows marked changes in gene expression between fetal day 17 and newborn, a period that encompasses the process of sacculation. Among the genes altered are the transcription factors Pod1 and GATA6, the stress-related gene Cyr61, surfactant protein D, and caveolin-1α. However, this important survey was not designed specifically to study sacculation, which would require sampling at more frequent time points near term.

As an alternative approach for understanding the molecular regulation of sacculation, it is reasonable to compare lungs with normal alveolar sac formation to those with altered sac formation and infer from differences in gene expression a set of candidate genes involved in the process. Preliminary expression microarray analysis of the GR knockout lung [16] has explored the overall changes in gene expression that lead to alterations in alveolar sac formation. This approach has also been used to study the related process of alveologenesis, that involves formation of septae that 'subdivide' alveolar sacs into smaller units or true alveoli [17].

Here, we use a microarray approach to begin to understand the molecular mechanisms by which formation of alveolar sacs is altered in T1α null mice, animals that serve as a highly reproducible model of altered lung sacculation. We have identified genes that are preferentially altered in the T1α null lung prior to recognizable structural abnormalities, such as ephrinA3, and identified that a number of regulators of the cell cycle are altered at term. Our study also shows that p21, a negative cell cycle regulator, is decreased at term but not at E18.5 at both the mRNA and protein levels, a finding that is likely associated with the failure of sacculation. These genes are now candidates for future studies designed to understand the molecular mechanisms that regulate sacculation in the normal lung.

## Results

### Altered alveolar sac morphology in the absence of T1α is evident on fetal day E18.5

**Figure 1:**
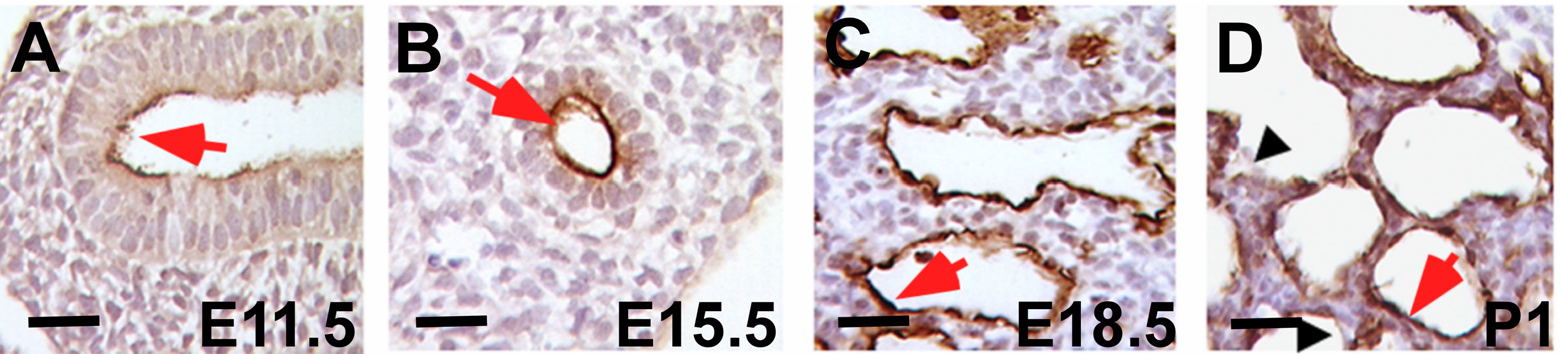
**Expression of T1α protein on the apical surface of mouse lung epithelial cells during development**. Immunohistochemistry analysis of T1α protein using mAb 8.1.1 antibody in mouse lung paraffin sections. (A) At E11.5 T1α is expressed on the apical surface of epithelial cells in the forming lung bud, (B) at E15.5 it is expressed in the cuboidal epithelial cells of the distal lung, (C) at E18.5 alveolar epithelial cells are starting to flatten and T1α antibody labels the apical surface of the alveolar walls, and (D) at postnatal day 1, type I cells express T1α protein while type II cells are not labeled. Red arrows indicate T1α expression. Black arrowheads indicate unstained type II cells at late gestation. Bar = 25 μm

**Figure 2:**
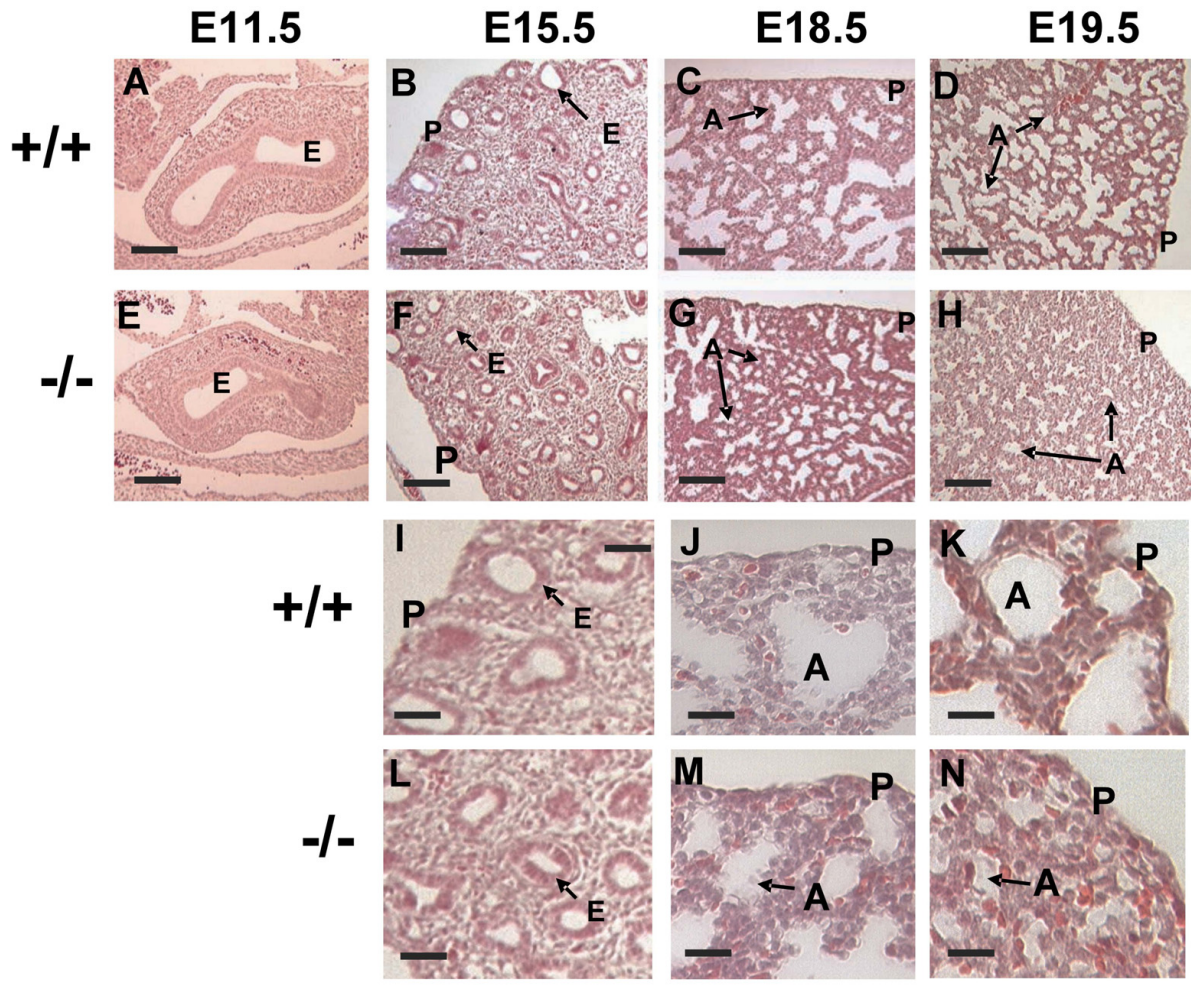
**Lung phenotype in T1α null mice is apparent after E15.5**. H & E staining of (A-D) T1α (+/+) and (E-H) T1α (-/-) lungs at E11.5, 15.5, 18.5, and 19.5 respectively Bar = 100 μm. (I-K) T1α (+/+) at E15.5, E18.5 and E19.5 (L-M) T1α (-/-) at E15.5, E18.5 and E19.5 Bar = 25 μm. Similar lung morphology is observed in T1α (+/+) and T1α (-/-) lungs at E11.5 and 15.5. The phenotype is more severe after E18.5. Thereafter, the lungs exhibit narrower airspaces and thicker mesenchyme. P, pleura; E, epithelium; A, alveolar space.

T1α protein is expressed in the apical surface of lung epithelial cells throughout mouse development (Figure 1). To determine the timing of the initial phenotypic alterations in the absence of T1α, we analyzed the distal lung morphology of T1α(+/+) and T1α(-/-) lungs at E11.5, 15.5, 18.5, and 19.5. Histological analyses showed no differences between T1α(+/+) and T1α(-/-) lungs at E11.5 and E15.5 (Fig. 2A,B,E,F). Gross morphology of the lobes and size of the epithelial buds are similar in (+/+) and (-/-) lungs at E11.5. No significant difference in epithelial tube number and size is apparent at E15.5 (Fig. 2I,L), suggesting that branching morphogenesis is not altered in T1α(-/-) lungs. In normal lung development, thinning of the mesenchyme, epithelial type I cell flattening, and enlargement of the air spaces are observed between E16.5 and term, as the alveolar sacs form. In the absence of T1α, the abnormal phenotype is evident at E18.5 and becomes progressively more severe at E19.5 (Fig. 2C,D,G,H) and at term [11], as the alveolar sacs are very narrow and irregular and the mesenchyme appears thicker than normal (Fig. 2K,N).

### Altered expression of PCNA and p21 genes

**Figure 3:**
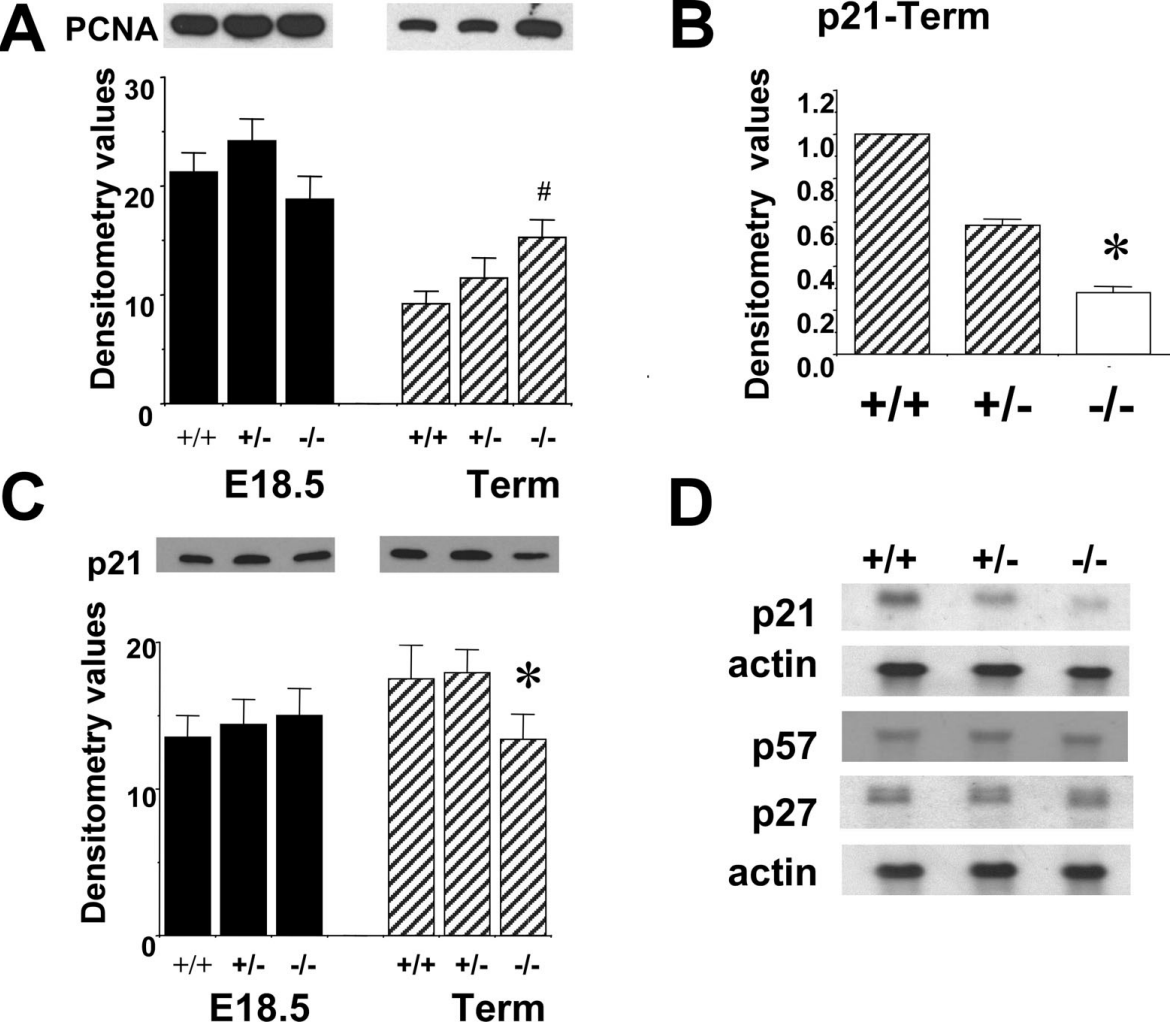
**p21 and PCNA expression is altered at term but not at E18.5 in T1α null lungs**. (A) Representative western blot and densitometry analysis of PCNA in total lung protein extracts (10 μg) at E18.5 and term, (B) densitometry analysis of p21 northern blots at term, (C) representative western blot and densitometry analysis of p21 in total lung protein extracts (10 μg) at E18.5 and term, and (D) representative blots of p21, p27 and p57 mRNA in total lung RNA at term (10 μg) normalized to β-actin. Time points analyzed were E18.5 and term. (+/+) indicates wild type, (+/-) heterozygous, and (-/-) homozygous genotypes respectively. n = 3 of each time point and genotype. Bars represent mean. Error bars represent Standard Deviation. (*) indicates p ≤ 0.05; (#) indicates p = 0.059.

To determine whether morphological alterations observed in the absence of T1α at E18.5 are also accompanied by changes in cell proliferation, we compared expression of PCNA and p21 genes by northern and western blot analyses in (+/+), (+/-) and (-/-) lungs at E18.5 and term. Neither PCNA nor p21 protein level is altered at E18.5 in T1α(-/-) lungs compared to T1α(+/+) (Fig. 3A–C). However at term, p21 mRNA is reduced 3.6-fold in (-/-) lungs (p ≤ 0.05) (Fig. 3B) and p21 protein is reduced to 75% of the level in (+/+) lungs (p ≤ 0.05) (Fig. 3C). We have previously shown high numbers of PCNA-stained cells at term, and that PCNA protein is significantly up-regulated ~1.8 fold in (-/-) lungs [11]. This trend is also observed in Fig. 3A (~1.7 fold, Student's t-test p = 0.059). The mRNA levels of other two members of the cdk inhibitor family, p27 and p57 at term are not altered (Fig. 3D). These data suggest that the increase in distal lung cell proliferation in the absence of T1α occurs after morphological alterations are evident, and is probably the consequence of earlier changes in gene expression triggered by the absence of T1α. In the microarray analysis neither p21 nor p27 are detected as present (detection p > 0.05) precluding the comparison to the northern blot analysis. PCNA and p57 are both detected as present in the microarrays (detection p ≤ 0.05) and their levels of expression do not change in the absence of T1α (-/-) compared to (+/+) at E18.5 or at term (data not shown), similarly to the data obtained by northern blots (Figure 3).

### Changes in gene expression in T1α null mutant lungs

To identify genes potentially involved in the abnormal alveolar sac formation of the T1α null lungs we analyzed global gene expression profiles of T1α(-/-) vs. T1α(+/+) lungs, at E18.5 and at term, using oligonucleotide microarrays. The data were analyzed using Student's t-test and the more stringent Mann-Whitney analysis [18]. We identified 239 genes that differ between (-/-) and (+/+) lungs at E18.5, with Student's t-test p ≤ 0.05, and median detection p value ≤ 0.05 at least in one genotype (Table S1, Additional file 3). Of those, 156 genes have a Mann-Whitney p value ≤ 0.05 (Table S3, Additional file 3). Genes in this group that are up (+) or down (-) regulated more than 1.5 fold with a Student's t-test p ≤ 0.05 and Mann-Whitney p ≤ 0.05 are shown in Table 1. At term, there are 50 genes that change significantly with a Student's t-test p ≤ 0.05 (Table S2, Additional file 3), Mann-Whitney p = 0.08 (Table S4, Additional file 3). The ability to detect genes that change significantly between T1α (-/-) and (+/+) at term was limited by the smaller sample size at that time point (n = 3). Known genes in this group changing more than 1.5 fold are shown in Table 2. The microarray quality control parameters analyzed, described in the Methods section, indicate that all hybridization experiments are comparable and of good quality.

**Table 1:** Genes differentially expressed in T1 α(-/-) vs T1 α(+/+) lungs at E18.5.

	Microarrays				
				
Gene	Fold	p	Acc #	Function	Lung expression and References
***Transcription factors***					
Sp1	-1.82	0.01	X60136	regulation of transcription	(+) (E1)
Sox11	-1.64	0.05	AF009414	regulation of transcription	(+) (E2)

***Signal transduction***					
Insulin-like growth factor I	-1.92	0.016	X04480	anti-apoptosis, organogenesis	(+) (E3, E4)
Sp 17	-1.82	0.008	Z46299	cAMP-dependent protein kinase regulator	(+) (E5, E6)
PKC delta	1.50	0.026	X60304	protein serine/threonine kinase activity	(+) (E7)
Signal-induced proliferation assoc.1	1.72	0.011	D11374	regulation of cell cycle	(+) UniGene Mm.3072
G protein gamma 3 linked	1.90	0.005	AF069954	RNA transport	(+) UniGene Mm.345134
Pip5k2c	2.35	0.005	AV303514		(+) UniGeneMm.22682

***Cytoskeleton/ECM***					
big-h3	-1.50	0.006	L19932	cell adhesion	(+) (E8)
dynein, cytoplasmic	1.58	0.007	AF063229	microtubule-based movement	(+) (E9)

***Ion channels/transport***					
Chloride channel regulator Icln	-1.86	0.03	U72059	chloride transport, regulation of cell volume	(+) (E10)
Solute carrier family 31	1.50	0.002	AI839005	copper ion transporter	(+) Unigene Mm.248637

***Enzymes***					
YME1-like	-2.33	0.008	AF090430	metalloendopeptidase activity	(+) Unigene Mm.23335
Holocytochrome c synthetase	-2.01	0.009	NM_008222	lyase	(+) MGI:106911
Cytochrome P450, 51	-1.81	0.011	AW122260		
Splicing factor 3a1	-1.69	0.044	AW120546	nuclear mRNA splicing, via spliceosome	(+) UniGene Mm.156914
Phosphodiesterase 7A	-1.62	0.0095	U68171	metal dependent phosphohydrolases	(+) (E11)
Ring finger protein 13	-1.62	0.008	AF037205	ubiquitin-protein ligase activity	(+) UniGene Mm.274360
Ptgs1	-1.54	0.008	M34141	prostaglandin biosynthesis	(+) (E12)
Paraoxonase 2	1.50	0.02	L48514	arylesterase activity	(+) (E13)
Protease serine 3	1.53	0.034	AE000665		n.d.
Cytochrome P450 4v3	1.57	0.006	BC026957		(+) UniGene Mm.245297
Branched chain aminotransferase 2	1.66	0.013	AF031467	amino acid biosynthesis	(+) UniGene Mm.24210
Neuropsin	1.74	0.011	D30785	serine-type endopeptidase activity	(+) (E14)
Catalase 1	1.80	0.016	AV083603	response to oxidative stress	(+) (E15)
Thioether S-methyltransferase	1.99	0.012	M88694	methyl transferase activity	(+) (E16)

***Immune system***					
Histocompatibility 2, D1	1.54	0.002	M69069	antigen presentation	(+) UniGene Mm.33263
Complement C1qa	1.82	0.004	X58861	complement activation	(+) UniGene Mm.370
Complement C1q B chain	1.82	0.023	M22531	complement activation	(+) UniGene Mm.2570

***Ubiquitination/Degradation system***					
Proteasome (macropain) alpha 2	-2.08	0.046	X70303	ubiquitin-dependent protein catabolism	(+) UniGene Mm.252255
Tetratricopeptide repeat gene	-1.75	0.029	AJ002730		(+) (E17)
Ube2v2	-1.58	0.022	NM_023585	ubiquiting conjugating enzyme activity	(+) UniGene Mm.235407

***Transcription/Translation/Protein synthesis***					
Eukaryotic translation initiation fac.2A	-2.15	0.017	AW061243	regulation of protein biosynthesis	(+) UniGene Mm.196220
FK506-binding protein (FKBP23)	-1.82	0.011	AF040252	peptidyl-prolyl isomerase	(+) (E18)
Eukaryotic translation initiation fac.1A	-1.72	0.036	AI132207	translation factor activity	(+) UniGene Mm.262037

***Cell Adhesion/Migration***					
Vascular cell adhesion molecule 1	-1.52	0.032	M84487	cell-cell adhesion	(+) (E19)

***Apoptosis***					
Programmed cell death 4	-1.54	0.014	D86344	isomerase	n.d.

***Cell-cell interaction***					
Sema4b	-1.69	0.047	AA266467	cell differentiation, development	n.d.
Ephrin B2	-1.64	0.011	U30244	development, neurogenesis, organogenesis	(+) (E21)
Ephrin A3	1.50	0.006	U92885	cell atraction-repulsion	(+) UniGene Mm.331159

***Other***					
Exportin1/CRM1 homolog	-2.00	0.009	AW123788	protein-nucleus export	(+) UniGene Mm.217547
NMDA receptor-regulated gene 1	-1.92	0.021	AW260482	angiogenesis, cell differentiation	(+) UniGene Mm.275281
Smarca5	-1.83	0.05	AA794509	chromatin remodeling	(+) (E22)
NP220	-1.81	0.008	D83033	RNA binding	(+) UniGene Mm.132392
Angiomotin	-1.78	0.042	AI854771	cell migration	(+) UniGene Mm.100068
IL13 receptor alpha 1	-1.67	0.02	AA608387	cytokine receptor activity	(+) UniGene Mm.24208
Kidney cell derived transcript	-1.69	0.008	U13371	myoD family inhibitor domain containing	(+) UniGene Mm.1314
Single strand DNA binding protein	-1.69	0.016	AA881160	DNA replication	n.d.
Rnpc2	-1.61	0.018	AA688834	transcription co-activator	(+) UniGene Mm.153895
ZAP3	-1.61	0.008	AB033168	differentiation	(+) UniGene Mm.153183
ADP-ribosylation-like factor 6 IP2	-1.57	0.02	AA763874	GTP-binding protein	(+) UniGene Mm.175403
Phosphatidylinositol glycan, class A	-1.59	0.025	D31863	GPI-anchor byosynthesis	(+) UniGene Mm.3781
Quaking	-1.56	0.04	U44940	vasculogenesis, locomotory behavior	(+) UniGene Mm.262294
WSB-1	-1.52	0.023	AF033186	signaling	(+) UniGene Mm.307022
Sorting nexin 17	1.51	0.037	AW123761	receptor mediated endocytosis	(+) UniGene Mm.6118
Disabled homolog 2 interacting protein	1.60	0.03	AI837497	GTPase activating protein	(+) UniGene Mm.29629
Zinc finger protein 64	1.73	0.026	U49046	development, transcription	(+) UniGene Mm.2095
Ribosomal protein L22	1.77	0.004	AI853960	ribosome biogenesis	(+) UniGene Mm.259907

Student's t test ≤ 0.05, Mann-Whitney p ≤ 0.05, detection p ≤ 0.05; UniGene = Expression profile suggested by analysis of EST counts; (+) lung expression

Microarray n = 5, Fold negative = down-regulated genes; Fold positive = up-regulated genes. nd.= no detected; (E) references in supplemental material

**Table 2:** Genes differentially expressed in T1 α(-/-) vs T1 α (+/+) lungs at term.

	Microarrays				
				
Gene	Fold	p	Acc #	Function	Lung expression and References
***Transcription factors***					
Egr1	-3.6	0.013	M28845	early response gene	(+) (E23, E24)
Nr4A1/Nur77	-2.4	0.050	X16995	modulation of RA signaling	(+) (E25, E26)
FosB	-1.6	0.023	X14897	regulation of cell cycle	(+) (E27)
Rb1	-1.5	0.048	AV338260	negative regulation of cell cycle	(+) (E28)

***Signal transduction***					
MKP-1	-1.6	0.042	X61940	MAPK phosphatase; cell cycle	(+) (E29)
PKCα binding protein	1.5	0.043	AV056986	kinase activity	n.d.

***Cell signaling***					
Cyr61	-2.8	0.0009	M32490	cell adhesion; growth factor bp	(+) (E30)

***Ion channels/transport***					
Cation channel C6	-2.0	0.030	U49069	Ca^2+ ^transport	(+) (E33)
ATPase, H+ transp. V1B2	-1.6	0.011	U13838	Cl^-^, H^+ ^transporter; ATPase	(+) (E34)

***Enzymes***					
Leucine aminopeptidase 3	-1.6	0.049	AI839225	proteolysis and peptidolysis	n.d.

***Immune system***					
IFN activated gene 205	-1.8	0.048	M74123		n.d.

***Other***					
S100a9	-1.9	0.035	M83219	calcium ion binding	(+) (E35)
Tumor differentially expressed 2	-1.5	0.025	AI834772		(+) UniGene Mm.29344
Additional sex combs like 1	1.8	0.032	AI852340	regulation of transcription	n.d.
Gas5	3.0	0.007	AI849615	negative control of cell growth	(+) (E36)

Student's t ≤ test 0.05, Mann-Whitney p = 0.08, detection p ≤ 0.05; n.d. not determined; (+) lung expression

Microarray n = 3; Fold negative = down-regulated genes; Fold positive = up-regulated genes; (E) references in supplemental material

We used EASE bioinformatic tool to identify over-represented biological processes, defined by the Gene Ontology database (GO), for 239 genes that have altered expression at E18.5 and for the 50 genes altered at term in the absence of T1α (data not shown). Genes involved in vasculogenesis, protein targeting, protein metabolism and mitosis are over-represented at E18.5 with an EASE score ≤ 0.05. Apoptosis, cell proliferation and regulation of transcription are over-represented at term with an EASE score ≤ 0.05. This analysis reflects the molecular events that occur in fetal and term lung in the absence of T1α during alveolar sac formation, and supports the finding that altered proliferation is a late gestation event that may be the result of earlier changes in gene expression in the absence of T1α. These data also correlate with the increased PCNA and reduced p21 protein expression observed at term. Changes in gene expression between T1α(-/-) and T1α(+/+) lungs either at E18.5 or at term are significant (p ≤ 0.05; not corrected for multiple comparisons) but moderate, considering the major morphological changes observed in the absence of T1α. We previously showed that the absence of T1α alters differentiation of type I epithelial cells but does not appear to affect other cell types of the distal lung at term, such as type II cells or endothelial cells [11]. Also, a similar proportion of epithelial to mesenchymal cells in T1α(-/-) and (+/+) lungs is suggested by unchanged levels of expression of the epithelial markers cytokeratin 8 and 18 and the mesenchymal marker collagen 1α1 at E18.5 and term (Figure S1, Additional file 1). Thus, molecular changes in an epithelial cell population that accounts for ~10% of the cells of the lung are likely to provide minimal signals when analyzed in the context of total lung mRNAs or proteins. These small but statistically significant changes are likely to be biologically relevant. Likewise, the molecular alterations in the absence of T1α could be at the post-transcriptional level.

**Figure 4:**
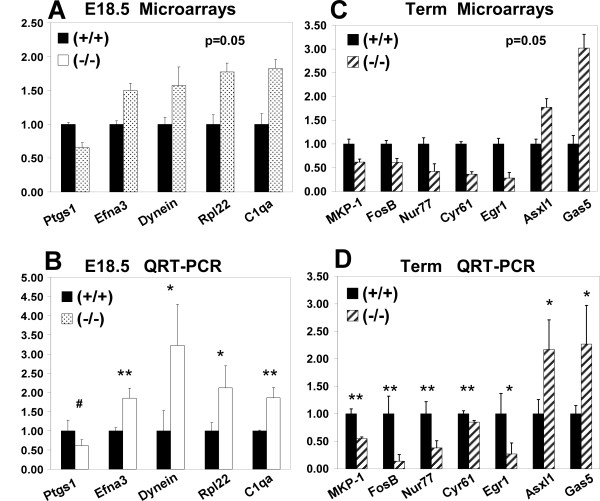
**Real time RT-PCR validation of selected genes identified by microarray analyses in E18.5 and term T1α null lungs**. **A**. Expression levels obtained by microarray analysis of RNA from E18.5 wild type (+/+) lungs (relative value = 1, black bars) compared to E18.5 T1α (-/-) lungs (fold change relative to wild type, dotted bars) (n = 5). **B**. Real time RT-PCR validation of the genes depicted in A (n = 3-6). **C**. Expression levels obtained in the microarray analysis of RNA from term wild type (+/+) lungs (relative value = 1, black bars) compared to term T1α (-/-) lungs (fold change relative to wild type, hatched bars) (n = 3). **D**. Real time RT-PCR validation of the genes depicted in C. Data is normalized to actin expression level. (n = 3-6). Error bars represent standard error of the mean. (*) indicates p ≤ 0.05; (**) indicates p ≤ 0.01; (#) indicates p = 0.056.

We evaluated by real time RT-PCR (QRT-PCR) changes in expression of selected genes identified by the microarray analyses (Figure 4). We confirmed the trend in expression observed in the microarrays for several genes, making them good candidates for further studies. Many of the genes have been previously described in the lung (see references for Tables 1 and 2, Additional file 2, or the UniGene database,[19]), and are therefore potentially involved in the formation of functional alveolar sacs.

**Figure 5:**
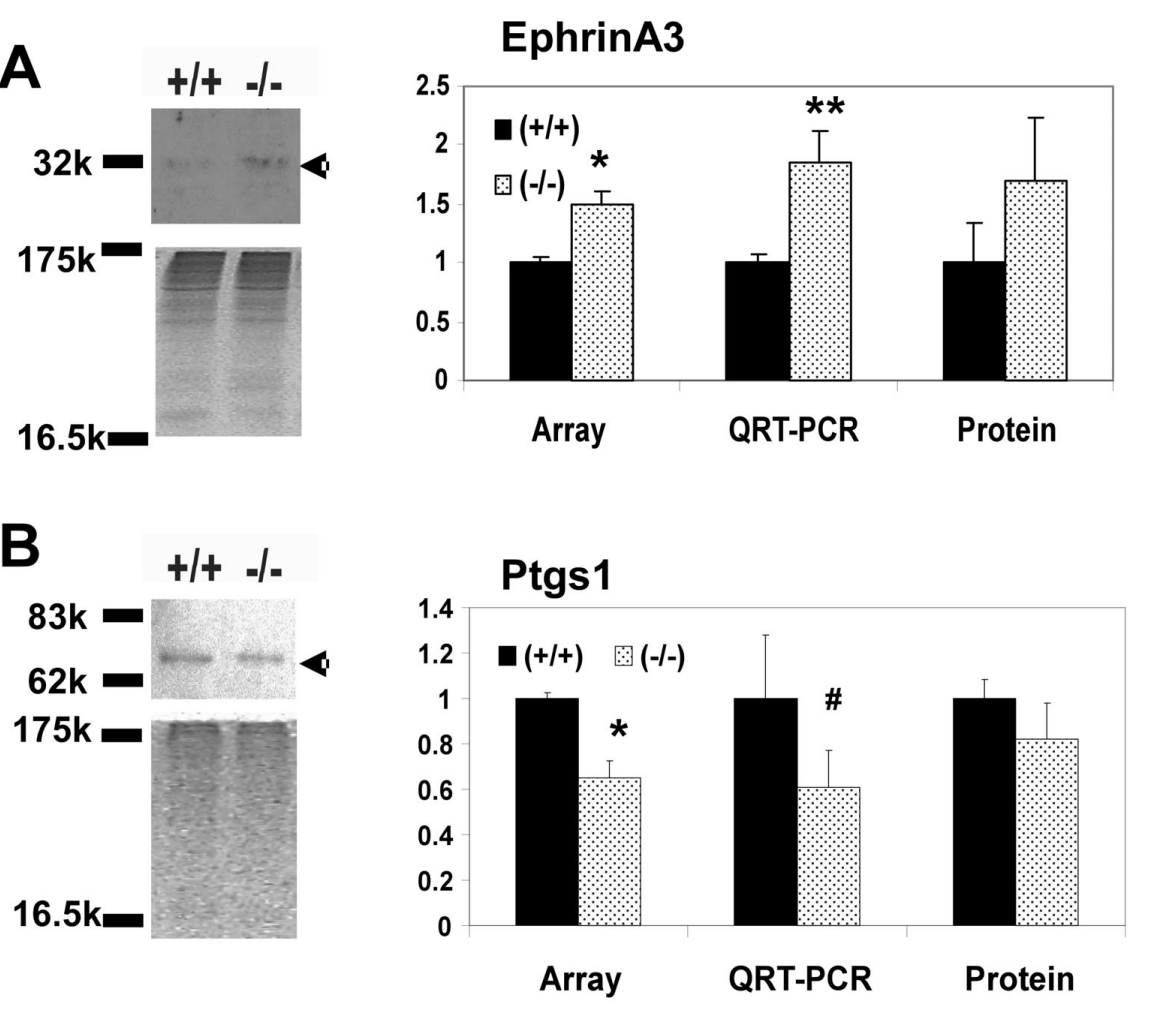
**Comparative expression analyses of mRNA and protein levels of EphrinA3 and Ptgs1**. Representative western blot and densitometry analysis of (A) EphrinA3 and (B) Ptgs 1 expression in total lung protein extracts (50 μg) at E18.5. Protein expression data are compared to the respective mRNA level determined by microarrays and QRT-PCR. Western blot data were normalized to the densitometry value of the Coomasie blue stained gels and total protein loaded. (+/+) indicates wild type, and (-/-) null genotypes respectively. n = 5 (E18.5) or 3 (term) for microarrays, n = 3 for QRT-PCR, and for western blot analyses for each genotype. Error bars represent standard error of the mean. (*) indicates p ≤ 0.05, (**) indicates p ≤ 0.01, (#) indicates p = 0.056.

At E18.5 we identified genes that are involved in cell-cell interaction, cell motility, prostaglandin metabolism, and signal transduction (Table 1). One of the genes involved in cell-cell interaction and cell motility is ephrinA3, which is up regulated at the mRNA and protein level (Figures 4A,B and 5A). Another member of the ephrin family, ephrin B2, is down regulated (Table 1). Cytoplasmic dynein, a motor protein involved in microtubule-mediated forward cell migration [20], is up regulated in the absence of T1α (Figure 4A,B). Genes involved in cell adhesion are also altered, such as the extracellular matrix protein βig-h3, and VCAM1 (Table 1). Ptgs1 (prostaglandin synthase 1; Cox-1) mRNA and protein expression levels are reduced in the absence of T1α (Table 1, and Figure 5B). Overall these changes in gene expression suggest that a different reorganization of the cellular structure is taking place in (-/-) lungs compared to wild type at E18.5.

At term, expression of several genes involved in regulation of cell proliferation is altered (Table 2). Among these, the early response genes FosB, Egr1, MPK-1 and Nur77 are down regulated (Figure 4C,D).

Some genes tested by QRT-PCR showed a variable level of expression in different T1α(-/-) lungs, such as Sp1 and Rb (data not shown). This is likely due to differences in the penetrance of the phenotype [11] within independent samples isolated in different experiments. The down-regulation of the expression of Cyr61 mRNA observed in the microarray analysis was not validated by other methods and Cyr61 protein level is significantly up-regulated in the absence of T1α (data not shown). This finding underscores the fact that changes in mRNA levels do not always correlate with changes in protein expression.

## Discussion

We have used the T1α knock-out mouse lung as a model of failed alveolar sacculation to begin to discover what genes are involved in the regulation of this critical morphogenetic event before birth. We previously reported that at term, lungs of the T1α knockout mouse display a severe phenotype [11], with airspaces that are small and often tortuous. The small airspaces cannot be expanded under positive pressure, indicating that the peripheral lung surface area is underdeveloped. Despite this dramatic phenotype, the null lungs at term have many normal features, including a normal number of type II alveolar epithelial cells, normal expression levels of surfactant protein mRNAs (SP-A, -B, -C, and -D), of a bronchiolar cell marker (CCSP), and of selected growth factors including FGF7 and FGF10 [11]. Of the genes tested, only aquaporin 5, another type I cell membrane protein, is reduced at both the mRNA and protein level. PCNA protein, a marker of post-proliferative cells, is increased at birth as was expected given the hyperproliferative phenotype of the term animals.

Several other genetically altered animals have shown a failure of sacculation resulting in lung histology that resembles the T1α deficient mice. The genes targeted in these animals therefore become candidates for key players in the process of sacculation. These include the transcription factors Sp3 [6] and NF1B [21], hormones and hormone receptors CRH [5] and GR [4], and inhibitors of the cell cycle p21/p57 (double p21(+/-)p57(+/-) mice) [7]. Mice deficient in these genes show failed sacculation at term, respiratory distress at birth, and usually neonatal death, while maintaining many normal features of the peripheral lung. The targeted genes that result in dysmorphic sacculation encode proteins of different functional categories, such as transcription factors or membrane receptors. It is noteworthy that null mutations of two important alveolar epithelial proteins, T1α and gp330/megalin [8,11], expressed in the apical membranes of type I and type II cells respectively, disrupt sacculation. T1α loss inhibits formation of expanded saccules, while loss of gp330/megalin results in abnormally enlarged saccules with cystic or emphysematous-like features [8].

While the identity of the genes knocked-out in the above animals is obviously known, none of these models of altered sacculation has been explored in detail to define the molecular pathways that lead to their phenotype. T1α, Sp3, GR, CRH, and megalin, for example, are all expressed early in lung embryogenesis and in the lung thereafter [14,22-25]. In those knock-outs there is considerable uncertainty about the timing of molecular events that, in the end, affect sacculation. One could envision that the accumulation of small molecular anomalies over time might block a major morphogenetic event. To address this question, we show here that the global gene expression patterns of T1α null lungs at gestational day 18.5 and term differ both from controls and from each other.

Comparison of microarray analyses of T1α-null and wild-type lungs on day 18.5 shows changes in expression of genes in several functional categories including transcription factors, cell-cell interaction, enzymes, channels, and others, that are reflected in overrepresentation of several biological processes including vasculogenesis, protein targeting, protein, metabolism, and mitosis. Among the cell-cell interaction genes ephrinA3 is significantly up-regulated in lungs showing narrow alveolar saccules. Ephrins are surface bound proteins that interact with eph tyrosine kinase receptors at sites of cell-cell interactions. The bidirectional signaling generated by this interaction regulates intercellular adhesion, cell shape and cell motility [26]. Ephrins, semaphorins, netrins and slit protein families generate molecular cues to guide cell movement and tissue morphogenesis [[27] and references therein].

Semaphorins, netrins and slit proteins appear to participate in early lung branching morphogenesis. In contrast there is sparse information about the role of ephrins in the lung. Several ephrinAs and ephA receptors are expressed in adult lung suggesting that they can also participate in cell-cell interaction in this organ. In particular ephrinA3 is highly up-regulated in squamous lung cell carcinomas [28]. The role of EphrinAs has been mostly studied in neurons and vascular formation [29,30] but it is possible that increased ephrinA3 in T1α(-/-) lungs alters the motility and/or shape of distal lung cells resulting in abnormal alveolar sac morphogenesis, a possibility that will require further evaluation.

Several types of enzymes such as peptidases, ligases, and others are affected at E18.5. One enzyme of particular interest is Ptgs 1 (Cox-1) that is decreased in T1α knockout lungs. Ptgs1 catalyzes the production of PGE2 that is the major prostaglandin species produced in the lung. PGE2 is known to be a negative regulator of lung fibroblast proliferation in adult rodents. While many lung cells can produce PGE2, murine alveolar epithelial cells are immunoreactive for COX-1 protein and adult alveolar type II epithelial cells have been directly shown to produce PGE2. Although the pattern of expression of Ptgs1 in mouse fetal lung is not known [31], decreased epithelial-derived prostaglandins due to a near absence of flattened type I cells, might affect mesenchymal cells in term T1α(-/-) lungs [32,33].

Biochemical data at term show that PCNA levels are higher and p21 levels are lower than normal in T1α(-/-) lungs. Microarray analyses of term T1α(-/-) lungs identify additional genes likely involved in the hyperproliferation phenotype of the term peripheral lung, such as FosB, Egr1, MKP-1 and Nur77. These immediate early genes are significantly down regulated. Changes in the expression of these genes were not observed at day 18.5. FosB belongs to the Ap-1 family of proteins regulated after mitogenic stimulation. c-jun/fosB dimers inhibit cell proliferation through binding to the Rb promoter [34]. Egr1 is a transcription factor responsive to a variety of stress and mitogenic signals that inhibits proliferation by activation of p21 [35,36].  Reduced expression of Egr1 in T1α (-/-) lungs may be involved in the down regulation of p21 transcription observed at term. The transcription factor Nur77 (NGFI-B) is an orphan nuclear receptor that modulates the retinoic acid signaling [37]. MKP-1 is a member of the dual specificity protein phosphatases that inhibits G1 progression in response to growth factors. Decreases in MKP-1 have been associated with increased proliferation [38]. MKP-1 transcription is up regulated by glucocorticoids through the GR receptor [39]. The similar phenotype of GR null lungs and T1α null lungs suggests that MKP-1 could be a common gene altered in these two mutant mice that regulates distal lung differentiation. Although all these changes point to possible abnormalities in cell cycle regulation, exactly how this might work and what the relationship between excess cell division and failure of sacculation is, remain unclear. Based on studies of other cells, decreased p21 would be expected to result in increased cell proliferation in the affected cell population(s). However because the net effects on cell cycle regulation are likely complex and dependent on the context of the affected cells it will be important in future studies to determine which lung cell types show alterations in proliferation related genes. It is possible that proliferation is repressed in some cell types while others are hyperproliferative, and are PCNA positive. An important site of decreased p21 expression is likely the type II cell which, in the T1α knock-out mouse, continues to express PCNA inappropriately near term. Although the sites of p21 expression are not well mapped in the fetal mouse lung, p21 and pro-SP-B proteins colocalize in newborn baboon lung under some conditions [40], and p21 mRNA is expressed in peripheral lung of fetal mice as shown by in situ hybridization [7]. If nascent type I cells are derived from post-mitotic type II cells, withdrawal from the cell cycle may be required before type I characteristics, including the distinctive morphology, can be expressed. This alone might affect sacculation. This possibility is consistent with the failed sacculation, neonatal respiratory death, and normal levels of surfactant proteins and CCSP in the double p21(-/-)p57(+/-) knockout mouse [7].

We have hypothesized that changes in gene expression might be few and of small magnitude based on previous northern analyses of null lungs at term [11]. Given the striking phenotype of the term lung, the relatively small change in many of the affected genes identified by microarray analysis, often between 1.5 and 2 fold, is notable. Many of these changes are highly significant, however, and are included in our assembled data because of the low variance between samples. Although a >twofold difference has been conventional in considering genes as functionally important, a number of previous studies have also opted to use lower cut-off levels (1.2–1.5) [41-43] when the changes are highly significant. In the T1α null lung this issue may relate in part to the fact that the type I cell comprises only about 10% of total lung cells; thus even large fold changes in genes expressed by type I cell precursors could be masked by lack of change of the same gene in other cell populations. In this regard it would have been useful to determine the fold changes in Aqp-5 mRNA by microarray because it is expressed only in type I cells in the peripheral lung; however Aqp-5 mRNA is not represented on the arrays we used.

## Conclusion

This global analysis of gene expression has identified a number of candidate genes that are significantly altered in lungs in which sacculation is diminished and has linked abnormal sacculation to alterations in the expression of cell cycle regulators. Some of these genes may be involved in the regulation of sacculation per se while others may be expressed as a consequence of failed sacculation. Our future studies will be directed towards further characterization of expression patterns genes identified, such as ephrinA3, Ptgs1 and the cell cycle regulators, as preliminary to test their functions in the sacculation process.

## Methods

### Animals

Generation of T1α null mutant [T1α(-/-)] mice was described previously [11]. T1α heterozygous [T1α(+/-)] mice were bred to generate timed pregnancies. The presence of a vaginal plug at noon was considered day 0.5 of pregnancy (E0.5). Fetuses were collected at E11.5, E15.5, E18.5 and E19.5. Term animals were obtained within 10 min of normal delivery. Mice were euthanized following IACUC-Boston University Medical Center approved procedures. Genomic DNA purified from a small piece of tail tissue was used to genotype the animals by PCR. Briefly, the targeting vector and/or endogenous T1α were detected using the following conditions: we amplified ~450 bp of the T1α promoter, absent in the targeted allele, using genomic DNA, Taq polymerase (QIAGEN, Inc., Valencia, CA), solution Q, 200 nM dNTPs, 94°C, 1 min; 50°C, 1 min; 72°C 2 min; 30 cycles; forward oligonucleotide (F) TGAATGAATGTAGGAGCGAGCTG; reverse oligonucleotide (R) TTAGGCTCTGAACCCACAATGG. We also amplified ~750 bp of the targeting sequence containing part of the 5' arm of homology and of the neo sequence using the same genomic DNA, Taq polymerase (QIAGEN), 1 mM MgCl_2_, 600 nM dNTPs, 94°C, 1 min; 60°C, 1 min; 72°C, 2 min; 30 cycles; forward oligonucleotide CCTTCTTGACGAGTTCTTCTGAGG; reverse oligonucleotide GGTCTTATCAGTTAGCTTGTGG.

### Histology and immunohistochemistry

E11.5 whole embryos or E15.5, 18.5, 19.5 and P1 dissected lungs were fixed in 4% paraformaldehyde/PBS pH 7.4 solution and embedded in paraffin as described previously [14]. Sections of T1α(+/+) and (-/-) lungs (6 μm) were stained with H&E to evaluate morphological changes in the absence of T1α as described previously [11]. T1α immunohistochemistry was performed as described by Kotton et al [44]. Briefly, antigen retrieval was performed by heating sections to 90°C in a citric acid buffer (antigen retrieval solution; Vector Laboratories) for 20 minutes, slowly cooling to room temperature, and prior to quenching. Sections were blocked with 1% goat serum in PBS (60 minutes), incubated overnight (4°C) with a monoclonal hamster anti-mouse T1α antibody (Developmental Studies Hybridoma Bank, University of Iowa, Hybridoma #8.1.1,[45]), and treated with HRP-labeled goat anti-hamster IgG (ICN, Costa Mesa, CA) for 30 minutes at room temperature. Signals were amplified using tyramide (TSA-Biotin System, NEN Life Science Products, Boston, MA) according to the manufacturer's protocol and exposed to diaminobenzidine for 6.5 minutes.

### RNA isolation, labeling and microarray hybridization

Total RNA from E18.5 and term T1α(+/+) and T1α(-/-) lungs was isolated (TRIzol method, Invitrogen, Carlsbad, CA), re-purified using RNeasy Mini Kit (QIAGEN), and DNase treated on column with DNA-free (Ambion, Inc., Austin TX). First and second strand syntheses were performed as recommended by Affymetrix using 8 μg total RNA as template. Biotin-labeled cRNA was generated using the ENZO BioArray High Yield RNA transcript labeling kit (Affymetrix, Santa Clara, CA). Each biotin-labeled cRNA sample (15 μg) was hybridized (16 hours at 45°C and 60 rpm) in an Affymetrix GeneChip System to the Murine Genome U74 Av2 GeneChip (Affymetrix) that contains ~12,000 sequences of the UniGene database or ESTs [46]. Confocal laser scanning was performed with a G2500A scanner (Agilent Technologies, Palo Alto, CA), to detect the streptavidin-labeled fluor.

### Microarray data acquisition

Five E18.5 T1α(+/+) lungs, five E18.5 T1α(-/-) lungs, three term T1α(+/+) lungs and three term T1α(-/-) lungs were analyzed on microarrays. A single weighted mean expression level for each gene was derived using Microarray Suite 5.0 software (MAS 5.0, Affymetrix). Using a one-sided Wilcoxon signed-rank test, the MAS 5.0 software generated a detection p-value for each gene indicating whether or not the transcript was reliably detected. Data from each array was scaled in order to normalize the results for inter-array comparisons.

To filter out arrays of poor quality, several quality control parameters on each array were assessed. We reviewed the scanned image for significant artifacts, and the presence on the array of bacterial genes spiked into the hybridization mix (bioB and bioC). Background and noise measurements were below 100 in all arrays. The average 3' to 5' ratio of the signal for GAPDH was 1.01 ± 0.15 and for β-actin was 1.63 ± 0.09 in E18.5 arrays. In term arrays, the 3' to 5' ratio for GAPDH was 1.63 ± 0.82 and for β-actin was 1.72 ± 0.54. A ratio less than 3 is considered acceptable. An average of 45.4 ± 3.8% probe sets exceeded the probe pair threshold in all arrays (called 'present'). Correlation values were calculated among arrays in the same genotype and developmental group. The average correlation values were: E18.5 T1α(-/-) (5 arrays) R^2 ^= 0.953 +/- 0.023, E18.5 T1α(+/+) (5 arrays) R^2 ^= 0.939 +/- 0.026, term T1α(-/-) (3 arrays) R^2 ^= 0.945 +/- 0.016, and term T1α(+/+) (3 arrays) R^2 ^= 0.931 +/- 0.026.

### Microarray data analysis

Prior to performing statistical analyses, we selected genes whose median detection p value was ≤ 0.05 (called 'present') in at least one of the 2 comparative groups (+/+ or -/-). To compare T1α(-/-) lung gene expression vs. T1α(+/+) at E18.5 (n = 5 each) or at term (n = 3 each), a Student's t-test was performed with a p-value less than 0.05 considered significant (Tables S1 and S2, Additional file 3). A Mann-Whitney test [18] was also performed given that expression levels of many genes did not follow a Gaussian distribution (Tables S3 and S4, Additional file 3). Due to the limited sample size, absolute correction for multiple comparisons was not performed. The magnitude of change in gene expression was calculated as the ratio of the average (arithmetic mean) signal in (-/-) lungs divided by the average signal in (+/+) at each time point and expressed as positive fold change (genes up regulated) or negative fold change (genes down regulated). Functional classification of differentially expressed genes was derived from the Gene Ontology annotation [47], and current literature. Microarray data have been deposited in the National Center for Biotechnology Information (NCBI) Gene Expression Omnibus (GEO) database [48]. GSE1363 is the GEO accession number for the series object that summarizes the experiments and provides links to all other relevant accessions [49].

Over-represented functional categories or "themes" altered in the absence of T1α at E18.5 and at term were identified using the EASE application [50]. EASE analyzes a list of Affymetrix ID numbers of the genes under study (input list) and finds over-represented biological "themes" in the list of genes, compared to the total number of genes for each biological theme in the array. The significance of each category is determined by two statistical values that are used to sort the categories, i.e. the standard Fisher exact probability, and a conservative EASE score that identifies robust categories. We considered categories with an EASE score ≤ 0.05 as over-represented.

### Real Time RT-PCR

Selected genes differentially expressed in the microarray analysis, not previously linked to alveolar sac formation, were evaluated by real time RT-PCR (QRT-PCR). E18.5 and term total RNA, treated with DNA-free DNase (Ambion), was reversed transcribed (RT) (1 μg in 25 μl) using AMV reverse transcriptase (Promega, Madison, WI). RT reactions diluted 1:32 were analyzed by QRT-PCR in an ABI 7000 instrument (Applied Biosystems, Foster City, CA). Primers were designed using PrimerExpress version 2.0 software (Applied Biosystems) or were obtained from Assays-on-Demand (Applied Biosystems). Reactions were performed in 50 μl using either TaqMan PCR universal master mix or SybrGreen PCR master mix (Applied Biosystems). For all primers and probes, optimal concentrations were found and dissociation curves were performed for primers used in SybrGreen reactions to confirm a single product. The relative concentration of RNA for each gene tested was obtained using calibration curves performed with wild type E18.5 or term total lung RNA (n = 3), respectively. Samples and calibration curves were reverse transcribed and amplified under equal conditions. E18.5 T1α (+/+), E18.5 T1α (-/-), term T1α (+/+), and term T1α (-/-) lungs were analyzed in duplicate (n = 3-6). Data were normalized to β-actin mRNA at E18.5 and at term; the level of expression of β-actin mRNA does not change between T1α(+/+) and T1α(-/-) samples at the respective time point. Oligonucleotides used in SybrGreen reactions were: Additional sex combs like (F) TCTTCCGCCATTTCATCTGA, (R) CACAACATGGACTTGGCCAA; Complement C1qa (F) GTGAAAGGCAATCCAGGCAA, (R) TGGTTGGTGAGGACCTTGTCA; Cyr61 Applied Biosystems Assays-on-Demand; Dual specificity phosphatase 1 (MKP-1) (F) ATGCTTGACACACCCACCAGT, (R) TCATACGCGCATGTCATCG; Dynein cytoplasmic (F) AAATCCACCCCAGCGTTTCTAC, (R) CCCCATTTGTTTTTGAGACGG; Early growth response 1 (F) TTTCAGCCTGAGTCCTTACCCA, (R) TGCATGGTCAGTTCCCAGAAC; Ephrin A3 (F) TGCCCCCTTCCTATGACAGAGAG, (R) GCCCAGAGAGATGTACAGTGCA; FosB (F) CGGCCTCTCCCTTTATCCTTT, (R) TGTCCCCGACAAATCCAGAA; Gas5 (F) TTCATTTGGCTGGCTTGCTT, (R) GGAATTCAAGGCCCCCAAT; Nuclear receptor subfamily 4A1/Nur77 (F) CCAGCCTGGTTTTGAGCTAAG, (R) CCATCTTCTGGAGTGAGGAGGT; OTS8/T1α (F) TGGCGTCTTGTTAGCCATTG, (R) GGCGAGACCTTCCAGAAATC; Prostaglandin-endoperoxide synthase 1 (F) CAAACCTACGTCTACGCCAA, (R) AGTCCATCTGTTCCCTCCAC; Rb1 (F) GAATTCTCAGAGATCTTGCTTCG, (R) AGTGGAGTGTCTGCAGCTTTAAG; Sp1 (F) CACCCAGTGCCAGAGACATATG, (R) CCAATTCTTCCTACCACCAAGC.

### Northern blots

Total lung RNA (10 μg) was electrophoresed, blotted and hybridized as described previously [11]. p21 probe was a gift of C. X. Deng, NIDDKD, NIH, Bethesda, MD). p27 and p57 probes were generated by RT-PCR (p27 forward oligonucleotide CGGGATATGGAAGAAGCGAGTC, reverse TTTACGTCTGGCGTCGAAGG; p57 forward oligonucleotide GCCAATGCGAACGACTTCTT, reverse AGAGTTCTTCCATCGTCCGCT). Data were normalized to β-actin [12].

### Western blots

E18.5 and term lung proteins were extracted using RIPA buffer [14]. Protein extracts (10–20 μg) were electrophoresed in a 12% SDS/polyacrylamide gel, blotted on PVDF membranes, or nitrocellulose for p57, and analyzed under similar conditions using PCNA, p21, p27, and p57 antibodies (Santa Cruz Biotechnologies, Inc., Santa Cruz, CA) as described previously [11]. EphrinA3 was analyzed in a 4% SDS/polyacrylamide gel, blotted on PVDF membranes and analyzed using ephrinA3 H-90 (1:1000) and cox-1 M-20 (1:500) antibodies (Santa Cruz Biothechnologies, Inc.). Histone H1 FL-219 (Santa Cruz), β-actin Clone AC-15 (Sigma, Saint Louis, MO) and tubulin-α (Sigma) were tested but discarded as potential controls for western blot loading because all three proteins show a higher expression in T1α(-/-) lungs compared to T1α(+/+) at term (data not shown). Therefore we used total amount of protein loaded as the normalization value, and Coomasie blue staining of the gel, where noted (n = 2-3, in triplicate).

## Authors' contributions

GM, has previously participated in the generation of the T1α null mutant mice. She obtained all the developmental lung samples for the studies and prepared samples for microarray hybridizations at the microarray core facility at Boston University. She also validated gene expression patterns by real time PCR; AH, performed histology and immunostaining analyses; JW, analyzed patterns of expression of cell cycle regulators by northern and western blot methods; AS participated in the design of the study, analyzed the microarray data, performed the statistical analyses, and provided his expertise to interpret the microarray data; MCW participated in the design of the study, in the interpretation of the data, and in the drafting of the manuscript; MIR participated in the design and coordination of the study, in the interpretation of the data, and in the drafting of the manuscript. All authors read and approved the final manuscript.

## Supplementary Material

Additional File 3 Supplementary Tables. Table S1. Genes dysregulated in T1α (-/-) vs. (+/+) lungs at E18.5 (p ≤ 0.05). Table S2. Genes dysregulated in T1α (-/-) vs. (+/+) lungs at term (p ≤ 0.05). Table S3. Mann-Whitney p values for genes dysregulated in E18.5 T1α (-/-) lungs compared to wild type, with Student's t test p ≤ 0.05. Table S4. Mann-Whitney p values for genes dysregulated in term T1α (-/-) lungs compared to wild type, with Student's t test p ≤ 0.05. Click here for file

Additional File 1 Figure S1. Ratios of epithelial vs. mesenchymal marker genes in T1α (+/+) and (-/-) lungs at E18.5 and term. Click here for file

Additional File 2 References in Table 1 and Table 2. The references include lung expression of specific genes. Click here for file
