# Supplementary material for: Alterations in gene expression in T1α null lung: a model of deficient alveolar sac development
Source: BMC Dev Biol. 2006 Jul 25;6:35. doi: 10.1186/1471-213X-6-35 (PMC1562362; doi:10.1186/1471-213X-6-35)
Supplement: Additional File 3 — Supplementary Tables. Table S1. Genes dysregulated in T1α (-/-) vs. (+/+) lungs at E18.5 (p ≤ 0.05). Table S2. Genes dysregulated in T1α (-/-) vs. (+/+) lungs at term (p ≤ 0.05). Table S3. Mann-Whitney p values for genes dysregulated in E18.5 T1α (-/-) lungs compared to wild type, with Student's t test p ≤ 0.05. Table S4. Mann-Whitney p values for genes dysregulated in term T1α (-/-) lungs compared to wild type, with Student's t test p ≤ 0.05. [file 1471-213X-6-35-S3.pdf]

Table S1. Genes dysregulated in T1α (-/-) vs. (+/+) lungs at E18.5 ($p \leq 0.05$)

Gene	Ratios (-/-)/(+/+) Array	Acc#
OTS8/T1α	0.12	M73748
**Transcription Factors**		
Sp1	0.55	X60136
sox11	0.61	AF009414
transcription factor IIE 2beta	0.78	AI844392
SCAN-KRAB zinc finger 1	0.80	AI852535
HNF-3/forkhead homolog 8	1.46	L35949
zinc finger protein 64	1.73	U49046
**Cell cycle**		
exportin1/CRM1 homolog	0.50	AW123788
centrin 2	0.59	AW047001
cdc2a	0.69	M38724
zac1 zinc finger protein	0.69	X95503
cdc 5-like	0.70	AI848968
cdc16	0.74	AW122965
p53-variant	1.25	U59758
**Signal transduction**		
CDC-like kinase	0.47	M38381
Sp 17	0.55	Z46299
phosphodiesterase7A	0.61	AI838731
disintegrin metalloprotease	0.62	AJ242912
GTP binding protein	0.63	U10551
dual-specificity tyrosine kinase 1a	0.68	U58497
erythropoietin 4	0.72	AW125150
ramp2	1.23	AJ250490
intersectin-EH binding protein Ibp2	1.29	AF057286
map2k3	1.29	AI852636
FMS-like tyrosine kinase 3	1.37	M64689
ramp1	1.46	AJ250489
PKC delta	1.50	X60304
gene rich cluster, C9 gene	1.60	AC002397
signal-induced proliferation assoc. 1	1.72	D11374
G protein gamma 3 linked gene	1.90	AF069954
pip5k2c	2.35	AV303514
**Cell signalling**		
insulin-like growth factor 1	0.52	X04480
bone morphogenetic protein 5	0.72	L41145
manic fringe	1.21	AF015769
dlk1-like homolog	1.53	Z12171
**Ion channels/Transport**		
chloride channel regulator Icln	0.54	U72059

ATP-binding cassette B7	0.62	AW124239
transmembrane 9 member 2	0.80	AI851289
4F2/CD98 light chain	1.29	AB017189
solute carrier family 4 , member 2	1.40	J04036

Enzymes

YME1-like (metalloprotease)	0.43	AF090430
holocytochrome c synthetase	0.50	AA645537
cytochrome P450, 51	0.55	AW122260
ring finger protein 13	0.62	AF037205
Ptgs1	0.65	M34141
glycogen phosphorylase	0.75	AI846739
guanylate kinase 1	1.19	U53514
FK506 bp9 (isomerase)	1.29	AF090334
arginosuccinate synthetase 1	1.30	M31690
alkaline phosphatase 2, liver	1.34	J02980
protective protein for b-galactosidase	1.36	J05261
amino levulinate synthase	1.37	M63245
phospholipase C, beta 1	1.44	U85714
UDP glucuronosyltransferase	1.47	U16818
paraoxonase 2	1.50	L48514
pyruvate carboxylase	1.56	M97957
branched chain aminotransferase 2	1.66	AF031467
neuropsin	1.74	D30785
catalase 1	1.80	AV083603
thioether S-methyltransferase	1.99	M88694
paraoxonase 1	2.00	U32684

Ubiquitination/Degradation system

proteasome (macropain) alpha 2	0.48	X70303
tetratricopeptide repeat gene	0.57	AJ002730
ubiquitin-conjugatingenzyme E2 v 2	0.63	AW060527
ubiquitin carboxyl-terminal esterase L5	0.66	AI838853
cullin 3	0.69	AI840051
proteasome, macropain, beta5	1.32	AB003304
ubiquitin-activating enzyme E1, Chr X	1.39	D10576

Immune system

Mac-2 antigen	1.24	X16834
toll interacting protein	1.41	AI842752
TAP binding protein	1.43	AI836367
T-cell death associated gene	1.54	U44088
histocompatibility 2, D1	1.54	M69069
complement C1q B chain	1.82	M22531
complement C1qa	1.82	X58861

Transcription/Translation/Protein synthesis

polymerase (RNA) II polypeptide G	0.44	AI836182
eukaryotic translation initiation fac. 2A	0.47	AW061243

FK506-binding protein (FKBP23)	0.55	AF040252
eukaryotic translation initiation fac. 1A	0.58	AI132207
splicing factor 3a sub 1	0.59	AW120546
splicing factor 3b sub 1	0.67	AI844532
SAP62-AMH	1.19	X83733
tryptophanyl-tRNA synthetase	1.25	AI851163
GTP binding protein (GEM)	1.29	U10551
polymerase (RNA) II polypeptide E	1.47	AI845735
**Cytoskeleton/ECM**		
SWI/SNF related	0.55	AA794509
chondroitin sulfate proteoglycan 6	0.59	Y15128
βig-h3	0.67	L19932
profilin 1	0.82	X14425
actinin alpha 4	1.25	AI836968
MAP/microtubule affinity reg. kinase	1.25	AI849218
septin 9	1.31	AJ250723
tubulin,alpha 8	1.36	AI848983
gelsolin	1.36	J04953
procollagen, type VI, alpha 1	1.38	X66405
cortactin	1.38	AI841808
vimentin	1.44	X56397
dynein, cytoplasmic	1.58	AF063229
**Tumor supressors**		
nore1	0.67	AF053959
**Oncogenes**		
ret-11	1.33	AB016784
**Cell Adhesion/Migration**		
vascular cell adhesion molecule 1	0.66	M84487
PEX2	0.76	AF031128
CEACAM1	1.27	X67279
CEACAM2	1.68	AF101164
retinal degeneration, slow	2.00	AV360626
**Apoptosis**		
PTEN	0.51	AI848984
programmed cell death 4	0.65	D86344
tia1	0.67	U00689
death-associated protein kinase 2	1.31	AB018002
BCL2-antagonist/killer1 (BAK)	1.37	Y13231
**Receptors**		
IL13 receptor alpha 1	0.60	AA608387
lymphotoxin B receptor	1.22	L38423
colony stimulating factor 1 receptor	1.38	X06368

Other

mitochondrial ribosomal protein L13	0.45	AA666635
fibroblast growth factor inducible 16	0.48	U42385
AZ2	0.49	AB007141
NP220 (nuclear protein)	0.55	D83033
D6Wsu176e	0.56	AA733372
lysosomal membrane glycoprotein 2	0.58	AI747194
single strand DNA binding protein	0.59	AA881160
maternal embryonic message 3	0.59	U47024
myotrophyn	0.60	D78188
ephrin B2	0.61	U30244
RNA binding region	0.62	AA688834
ZAP3 (nuclear protein)	0.62	AB033168
ADP-ribosylation-like factor 6 IP2	0.63	AA763874
phosphatidylinositol glycan, class A	0.63	D31863
ribosomal protein L23	0.63	U84903
quaking	0.64	U44940
WSB-1	0.66	AF033186
ash1l	0.67	AW121855
histone H3	0.70	M32459
nedd1	0.72	D10712
dynamin 1-like	0.74	AW120468
D7Wsu128e	0.77	AA388099
lymphocyte specific 1	0.81	AV122642
zinc finger protein 277	0.83	AW121594
histone 2A	1.07	AI853078
D13Wsu64e	1.18	AW047811
zygin II ortholog	1.23	AI851119
cog1	1.26	AF109377
D11Wsu68e	1.28	AI847163
inhibitor of growth 1	1.32	AA647507
cytochrome beta-558	1.33	M31775
mic2-like1	1.33	AW050035
seizure-related	1.35	D78643
calnexin	1.35	L18888
BS4 peptide	1.36	U27462
chaperonin subunit 7	1.36	Z31399
rex3	1.37	AA790008
PEDF	1.38	AF036164
vesicle associated membrane protein	1.39	AI849048
bladder cancer associated	1.48	AW121500
CTD small phosphatase 2	1.48	AV366282
ephrin A3	1.50	U92885
solute carrier family 31	1.50	AI839005
sorting nexin 17	1.51	AW123761
D8Wsu151e	1.54	AI852472
mitochondrial ribosomal protein S18A	1.58	AW230209
ribosomal protein S19	1.67	AW048899
ribosomal protein L22	1.77	AI853960

ESTs/RIKEN/IMAGE

EST AI853703	0.49	AI853703
EST AA409398	0.54	AA867340
EST C81439	0.56	AI854771
transcribed sequence	0.57	AI463656
D10Ertd438e	0.57	AI839117
RIKEN 1110055L24	0.58	AI853900
IMAGE-718528	0.59	AA266467
clone1.5	0.59	U13371
RIKEN 4921506J03	0.60	AW048484
RIKEN 2310035M22	0.61	AI851230
EST AW047583	0.62	AW047583
IMAGE-746435	0.66	AA260005
RIKEN 2610044B22	0.66	AI844034
DNA segment, Chr 3	0.67	AW060249
RIKEN 2010200I23	0.69	AW122573
EST	0.69	AA145282
IMAGE-1179231	0.70	AI645561
RIKEN 2810034J18	0.70	AW045597
RIKEN 1200003C05	0.70	AW046391
RIKEN 2500001H09	0.71	AA615429
RIKEN 9030416H16	0.71	AI854581
IMAGE-1122163	0.72	AA638002
RIKEN 1810046J19	0.73	AI852571
RIKEN 2610019N13	0.73	AW122195
EST Dynamin-1	0.74	AW120468
RIKEN 1110014E10	0.74	AA199023
RIKEN 1700016A15	0.75	AI197431
EST 2939292	0.76	AI850972
EST AA408298	0.77	AA388099
EST AI465155	0.80	AA644817
RIKEN 2310008D10	1.16	AW123754
EST AA408242	1.16	AW123404
RIKEN 0710007A14	1.17	AW122179
RIKEN 1110008F13	1.17	AI846102
RIKEN 5430432M24	1.18	AI845815
RIKEN 9530042F15	1.18	AI838053
IMAGE-3602181	1.20	AW050142
EST T17286	1.24	AI838576
RIKEN 2410112006	1.24	AI875598
D18Wsu98e	1.24	AI846519
RIKEN 2310075G12	1.25	AW049793
RIKEN 2610028J07	1.26	AW048053
EST AF006998	1.27	AW046336
RIKEN 3110038L01	1.28	AI844396
RIKEN 0610007O07	1.29	AI851762
IMAGE-1154478	1.30	AA796989
RIKEN 9030624B09	1.30	AI851614
RIKEN 0710001K01	1.32	AW122159
RIKEN 2810024B22	1.33	AW122429
RIKEN 1110006I15	1.34	AI853127
RIKEN 2810477h02	1.35	AW125010

RIKEN 1810006O10	1.36	AW047762
RIKEN 0610012F07	1.37	AW012359
EST AI414047	1.37	AW125649
RIKEN 1110035L05	1.38	AI839150
IMAGE-5135570	1.38	AW125222
RIKEN 1500001N04	1.41	AW061222
RIKEN 2310074H19	1.41	AI844737
RIKEN 1810031K02	1.44	AI850357
IMAGE-5065541	1.44	AI847092
IMAGE:3156885	1.47	AI845735
RIKEN C13052I12	1.47	AI83598
EST AI838661	1.50	AI838661
EST AI838661	1.50	AI838661
EST	1.53	AE000665
MGC38258	1.54	AW120628
IMAGE-574446	1.56	AI447510
transcribed sequence	1.57	AI642417
RIKEN 0610011I04	1.57	AI787183
EST AW111961	1.57	AA212964
EST AI480459	1.60	AI837497
EST BC005662	1.66	AW060889
RIKEN 2610005L07	1.84	AV346841
transcribed sequence	1.88	AI847033

Table S2. Genes dysregulated in T1α (-/-) vs. (+/+) lungs at term ($p \leq 0.05$)

Gene	Ratios (-/-)/(+/+) Array	Acc#
**Transcription factors**		
early growth response 1	0.28	M28845
nuclear receptor subfamily 4A1 / nurr77	0.42	X16995
FosB	0.61	X14897
Rb1	0.67	AV338260
nuclear factor I/X	0.71	Y07688
nuclear receptor subfamily 2F2 / Arp-1	0.74	X76653
trasformation related protein 53 bp1	1.19	AI593074
zinc finger protein 54	1.30	AF080070
interleukin enhancer binding factor 3	1.38	AI835460
**Cell cycle/proliferation**		
spindlin	0.70	AW122015
chromosome segregation 1-like	0.85	AW123099
**Signal transduction**		
dual specificity phosphatase 1 (MKP-1)	0.61	X61940
PKC alpha bp	1.54	AV056986
**Cell signaling**		
cyr61	0.36	M32490
fibroblast growth factor 7	0.57	Z22703
**Ion channels/Transport**		
transient receptor potential cation channel C6	0.51	U49069
ATPase, H+ transporting V1B2	0.62	U13838
**Enzymes**		
leucine aminopeptidase 3	0.64	AI839225
phosphatidylinositol-4-phosphate 5-kinase IIa	0.73	AI843864
gluthathione S-transferase, mu 5	0.78	U24428
small fragment nuclease	0.78	AI839882
GPI anchor attachment protein 1	1.12	AB002136
protein-L-isoaspartate O-methyltransferase1	1.18	AW124044
hydroxysteroid (17 beta) dehydrogenase 4	1.20	X89998
exostoses 2	1.21	U72141
3-ketoacyl-CoA thiolase B	1.27	AW012588
**Immune system**		
interferon activated gene 205	0.56	M74123
**Apoptosis**		
ring finger protein 7	0.71	AI843444
B-cell leukemia/lymphoma 10	0.80	AJ006289

cradd	0.81	AJ224738
**Receptors**		
dopamine receptor 2	0.73	X55674
Eph receptor A5	0.80	AI854630
**Other**		
S100 calcium binding protein A9	0.53	M83219
tumor differentially expressed 1, like	0.67	AI834772
mitofusin 1	0.72	AV255723
replication protein A3	0.75	AI848299
zinc finger protein	0.75	D10627
mitochondrial ribosomal protein L45	1.22	AI841415
meningioma expressed antigen 5	1.27	AI835427
additional sex combs like 1	1.77	AI852340
**ESTs/RIKEN/IMAGE**		
EST	0.71	AW046351
RIKEN 6030448M23	0.73	AA259683
RIKEN 2310005K03	0.83	AW018420
RIKEN 5830451P18	1.18	AI851751
RIKEN 1110059E24	1.25	AI854226
RIKEN 2810465O16	1.29	AI648758
RIKEN 5930412E23	1.35	AW120890
RIKEN 5730507C05	1.49	AI842878
EST C76800	1.55	AI853340
RIKEN D030018K05	3.02	AI849615

Table S3. Mann-Whitney p values for genes dysregulated in dpc 18.5 T1α (-/-) lungs compared to wild type with Student's t test p less 0.05.

affy id	Gene Symbol	t-test	mann-whitney	log t-test
93315_at	Map2k3	0.00041129	0.0121856	0.000585748
160897_at	BC005662	0.00096714	0.0121856	0.00033542
104469_at	Gp38	0.00117473	0.0121856	0.00744808
103845_at	Slc31a1	0.00222336	0.0121856	0.00564148
97540_f_at	H2-D1	0.00244797	0.0121856	0.00124206
104188_at	Notch2	0.00269316	0.0121856	0.0042931
94435_at	D10Ertd438e	0.00282198	0.0215718	0.00413575
96617_at	Drap1	0.0032677	0.0121856	0.00853238
96925_at	2810024B22Rik	0.00345059	0.0215718	0.00200038
92857_at	Rpl22	0.00366844	0.0215718	0.00669636
98562_at	C1qa	0.00375576	0.0121856	0.014543
160562_at	Cct7	0.00431779	0.0121856	0.00321649
161763_r_at	Pip5k2c	0.00455354	0.0121856	0.000635884
93080_at	Bscl2	0.00520848	0.0121856	0.0459932
160387_at	1110055L24Rik	0.00526935	0.0121856	0.00590237
104609_at	AI465155	0.00527725	0.0121856	0.00830813
92877_at	Tgfbi	0.00554323	0.0215718	0.0120622
94054_at	Cttn	0.00566406	0.0121856	0.00306134
100289_at	Efna3	0.00629231	0.0121856	0.00175208
160611_at	Cyp4v3	0.00642797	0.0121856	0.00429849
95002_at	D17Wsu92e	0.0065075	0.0121856	0.0165509
160251_at	2610312B22Rik	0.00653788	0.0215718	0.00396622
98544_at	Guk1	0.0067262	0.0121856	0.00489275
103809_r_at	Dncic1	0.00680433	0.0215718	0.00977107
95597_at	Ptgs1	0.00770261	0.0121856	0.0193669
93731_at	Fkbp9	0.00771664	0.0121856	0.0054016
99999_at	Np220	0.0078186	0.0215718	0.00509118
101850_at	Spa17	0.00782596	0.0215718	0.00513878
102644_at	Kdt1	0.00799231	0.0367141	0.0138284
160831_at	AI838661	0.0080381	0.0121856	0.00351813
104535_at	Yme1l1	0.00827221	0.0215718	0.00837918
101965_at	Rnf13	0.00843706	0.0121856	0.00314272
100413_at	Zap3	0.00853462	0.0121856	0.00672442
98609_at	Septin 9	0.00887165	0.0215718	0.0081953
160573_at	Hccs	0.00887773	0.0121856	0.0768029
100032_at	Aaas	0.0089605	0.0121856	0.00142671
96113_at	D18Wsu98e	0.00906593	0.0121856	0.00565049
94228_at	Xpo1	0.00921935	0.0121856	0.00273727
100489_at	Pde7a	0.00948563	0.0121856	0.00474074
93426_at	1110014E10Rik	0.00959311	0.0215718	0.0155724
104710_at	Bak1	0.00992029	0.0215718	0.0125196
161104_at	9430099J10Rik	0.0103834	0.0367141	0.0104756
94916_at	Cyp51	0.0106288	0.0367141	0.0102795
99327_at	Prss19	0.0109238	0.0121856	0.0270548
97963_at	Sipa1	0.0110316	0.0121856	0.0109849
104746_at	Fkbp7	0.0112765	0.0367141	0.00910328
160559_at	Psmb5	0.0113728	0.0215718	0.00779142
160857_at	Efnb2	0.0115169	0.0215718	0.030154

100570_at	6330412F12Rik	0.0115448	0.0367141	0.0153401
96219_at	1810031K02Rik	0.0115949	0.0215718	0.0181584
97521_at	Ass1	0.0116375	0.0215718	0.0133724
97402_at	Temt	0.0119489	0.0215718	0.0163194
104501_at	Vapb	0.0126864	0.0121856	0.0232096
100443_at	Bcat2	0.0127679	0.0367141	0.0175093
95480_at	D11Wsu68e	0.0131033	0.0215718	0.0219808
93627_at	Ankrd28	0.0136574	0.0121856	0.00694795
161004_at	1700097N02Rik	0.0137275	0.0215718	0.0143181
95666_at	Cops8	0.0139027	0.0215718	0.0162895
92796_at	Akp2	0.0141789	0.0215718	0.0134729
161879_r_at	Ctdsp2	0.0144521	0.0121856	0.00384731
104354_at	Csf1r	0.0149978	0.0367141	0.0203206
93021_at	LOC406217	0.0150866	0.0215718	0.0340482
94989_at	---	0.0152694	0.0215718	0.0128797
160479_at	Cat	0.015498	0.0215718	0.0107586
100957_at	Ssbp1	0.015742	0.0367141	0.0291077
96790_f_at	Galm	0.0157421	0.0282803	0.0176841
97922_at	Ncb5or	0.0160029	0.0367141	0.0141921
104310_at	G6pc3	0.0160558	0.0215718	0.0206132
95546_g_at	Igf1	0.0161813	0.0162936	0.0381631
104115_at	Psme4	0.0166867	0.0121856	0.00441204
160105_r_at	2810006K23Rik	0.0169273	0.0215718	0.0313833
96609_at	2610019N13Rik	0.017047	0.0367141	0.0212686
94253_at	Eif2s1	0.0174312	0.0215718	0.00461359
100572_at	Tm9sf2	0.0175544	0.0367141	0.0157951
102333_at	Epn2	0.0175754	0.0121856	0.0101612
103763_at	Ash1l	0.0176449	0.060103	0.0222018
97808_at	Sf3b1	0.0180069	0.0215718	0.0169344
100020_at	Slc4a2	0.0180376	0.0367141	0.024593
93267_at	Rnpc2	0.0182878	0.0367141	0.0401683
97517_at	Exosc4	0.01833	0.0121856	0.0114255
97770_s_at	D6Wsu176e	0.0184167	0.0367141	0.012049
94073_at	Polr2g	0.0194741	0.0121856	0.00300744
96236_at	Cdc16	0.0195964	0.0121856	0.0116638
103723_at	Il13ra1	0.0197684	0.0215718	0.0143598
95135_at	3110038L01Rik	0.0200823	0.0215718	0.0290796
104193_at	2810485I05Rik	0.0201011	0.0121856	0.00457972
104745_at	Arl6ip2	0.0205164	0.0367141	0.0254737
93246_at	Narg1	0.0208399	0.0367141	0.0189431
96605_at	0610011I04Rik	0.0209989	0.060103	0.0323103
99866_at	---	0.0217158	0.0367141	0.0335736
160247_at	Ube2v2	0.0217271	0.0367141	0.0287671
104682_at	Tuba8	0.0217695	0.0367141	0.019256
93750_at	Gsn	0.0219799	0.0367141	0.0346038
92797_at	Cul3	0.0220593	0.0367141	0.0177183
103808_at	Golga5	0.0220781	0.0215718	0.0346476
160378_at	1110006I15Rik	0.0220835	0.0367141	0.0222675
160829_at	Phlda1	0.0223923	0.0367141	0.0149868
95706_at	Lgals3	0.0225244	0.060103	0.0274093
98946_at	Wsb1	0.0227321	0.0367141	0.0378509

101295_s_at	Clns1a	0.0228018	0.0367141	0.0277578
96020_at	C1qb	0.0228039	0.0367141	0.036604
96152_at	Narg1	0.0229064	0.0367141	0.0190707
95613_at	2010200I23Rik	0.0229724	0.0367141	0.0223023
92466_at	Plcb1	0.0236524	0.0367141	0.014801
101000_at	Oaz2	0.0239603	0.0215718	0.0412118
96895_at	Pon1	0.0242943	0.060103	0.0425241
97226_at	Gna12	0.0243071	0.0215718	0.0215436
104680_at	Ramp1	0.0244665	0.0215718	0.0435234
92304_at	Piga	0.0245849	0.0121856	0.00667178
96070_at	Pfn1	0.0251043	0.060103	0.022253
101590_at	Lamp2	0.0252585	0.060103	0.0252681
94068_at	Rps19	0.0256569	0.060103	0.0321055
104378_at	Pon2	0.0257751	0.0121856	0.0313137
101934_at	Fez2	0.0259705	0.0121856	0.0153669
103047_at	Pxmp3	0.0259898	0.0367141	0.0508649
103841_at	Zfp64	0.0261159	0.0367141	0.0291443
160698_s_at	Prkcd	0.0262703	0.0367141	0.02344
103993_at	Grcc9	0.0263294	0.060103	0.0212953
101913_at	T25545	0.0267567	0.0367141	0.0331488
97446_at	Dhx30	0.0270596	0.060103	0.0229121
99149_at	2310035M22Rik	0.0274546	0.0215718	0.0664327
102028_at	Rassf5	0.0275687	0.0367141	0.0546214
97489_at	Pygb	0.028386	0.0367141	0.0344847
100059_at	Cyba	0.0284246	0.060103	0.0192512
92616_at	Ube1x	0.0286063	0.0367141	0.0271168
94396_at	Ing1	0.0287797	0.0367141	0.0426459
102222_at	Utx	0.0291308	0.0121856	0.0102119
98605_at	Wars	0.0292375	0.0367141	0.0275521
161462_r_at	---	0.0292541	0.060103	0.0301902
95161_at	Ctdsp2	0.0295371	0.060103	0.0163253
160302_at	1200003C05Rik	0.0295815	0.0946929	0.0538552
160333_at	1110008F13Rik	0.0296264	0.060103	0.0265347
97353_at	Dab2ip	0.0297732	0.0215718	0.0477383
160823_at	Nedd1	0.030337	0.0367141	0.0235341
97846_at	Cdc5l	0.0304767	0.060103	0.0449984
98849_at	D8Bwg1414e	0.0306796	0.047202	0.0368607
104221_at	Slc7a5	0.0308065	0.0121856	0.0177199
93274_at	Clk	0.0308176	0.0946929	0.0211471
95102_at	Scotin	0.0310644	0.060103	0.030027
161874_r_at	---	0.0310813	0.060103	0.0287354
98976_at	Adamdec1	0.0313796	0.060103	0.0251116
97829_at	Cdipt	0.0314656	0.0946929	0.0282249
98369_f_at	---	0.0315255	0.060103	0.0338837
99506_at	Dapk2	0.0316439	0.060103	0.0382641
92558_at	Vcam1	0.0318102	0.0367141	0.0260602
160791_at	3300001P08Rik	0.0318241	0.060103	0.0297063
101618_r_at	---	0.0319391	0.060103	0.0323482
96045_at	2010321M09Rik	0.0321515	0.0758003	0.0358572
162459_f_at	Col6a1	0.0322252	0.0367141	0.060372
160696_at	Tia1	0.032251	0.0367141	0.0233063

99444_at	Ramp2	0.0323163	0.0367141	0.0384337
97980_at	Ltbr	0.0324711	0.0946929	0.0383048
93574_at	Serpinf1	0.0324939	0.060103	0.0387547
98912_at	D13Wsu64e	0.0325442	0.047202	0.0243674
162379_r_at	Vim	0.0326575	0.060103	0.0304699
99393_at	Bmp5	0.0328217	0.060103	0.0265175
101975_at	Dlk1	0.0329311	0.060103	0.0494488
93439_f_at	Pawr	0.0332407	0.060103	0.0495186
101339_at	Try3	0.0336165	0.0215718	0.0228305
93500_at	Alas1	0.033982	0.060103	0.042314
160435_at	Mic2l1	0.0344767	0.0215718	0.0520968
100508_at	Mfng	0.0345612	0.060103	0.0328416
98933_at	Mark3	0.034565	0.060103	0.0262853
99580_s_at	Ugt1a2	0.0348694	0.060103	0.0210875
161914_s_at	Lsp1	0.0349662	0.0367141	0.04619
161683_r_at	Gtpbp1	0.035091	0.0946929	0.0304152
103300_at	Abcb7	0.0353684	0.060103	0.0476997
101055_at	Ppgb	0.035984	0.0946929	0.0392224
103708_at	Eif1a	0.0360915	0.0367141	0.0290896
94453_at	1810046J19Rik	0.0361038	0.0215718	0.0217836
96653_at	Rnaset2	0.0366468	0.0367141	0.0556572
161257_r_at	Snx17	0.0367795	0.0121856	0.0180087
102804_at	Ceacam2	0.0368405	0.060103	0.0457532
94983_at	1810073G14Rik	0.0373163	0.0367141	0.047087
96755_at	---	0.0374514	0.0367141	0.0298155
100154_at	Tapbp	0.0375484	0.060103	0.0395093
96343_at	Actn4	0.0378206	0.060103	0.0388786
102853_at	Cspg6	0.0378291	0.060103	0.0382819
160931_at	Dnm1l	0.0383809	0.060103	0.0354951
94937_at	Zfp277	0.039021	0.0946929	0.0420649
160726_at	Qk	0.0393771	0.0367141	0.0348655
160468_at	Mtpn	0.0395168	0.060103	0.0358356
104733_at	Cetn2	0.0398068	0.060103	0.12933
93472_at	AI413331	0.0400404	0.0121856	0.0272655
95296_r_at	Flt3	0.0401082	0.0367141	0.0436435
103595_at	2300009P13Rik	0.040224	0.0367141	0.0357595
100128_at	Cdc2a	0.0402412	0.0946929	0.0277946
161498_at	---	0.0404324	0.0946929	0.036541
97421_at	Smc2l1	0.0404831	0.0946929	0.0191972
100889_at	BC042901	0.0406971	0.0215718	0.0476904
160386_at	C130052I12Rik	0.0413904	0.0946929	0.0617652
98515_at	Gtf2e2	0.0413943	0.0946929	0.0353243
94327_at	Mrps18a	0.0414443	0.0758003	0.0802372
97452_at	H2afy	0.0416174	0.0367141	0.0376478
95531_at	Amot	0.0416428	0.0367141	0.164823
96750_at	0710007A14Rik	0.0418649	0.0367141	0.0457727
96270_at	D11Bwg0434e	0.0419206	0.0946929	0.0459089
95432_f_at	D16Wsu109e	0.042174	0.060103	0.0338019
102982_at	B230308N11Rik	0.0423723	0.060103	0.0338622
96327_at	Skz1	0.0425992	0.0946929	0.0564423
100512_at	Uchl5	0.0427289	0.0946929	0.0701556

92196_f_at	Sf3a2	0.0428057	0.060103	0.0425054
104453_at	2310079P12Rik	0.0429584	0.143672	0.0670433
101908_s_at	Ceacam2	0.0432535	0.060103	0.050078
99923_at	5730417B17Rik	0.0434367	0.0946929	0.0556633
96027_at	Sf3a1	0.043973	0.0367141	0.0194929
160809_at	Tollip	0.0440936	0.0946929	0.0491735
95449_at	2310075G12Rik	0.0441824	0.0946929	0.0436504
103343_at	5430432M24Rik	0.0442608	0.060103	0.0438393
103029_at	Pdcd4	0.0444633	0.060103	0.0413157
102019_at	Mrpl13	0.0445799	0.060103	0.0229154
94886_at	Canx	0.04462	0.0946929	0.0537644
92501_s_at	Plagl1	0.0447635	0.0946929	0.0370062
93023_f_at	Hist2h3c2	0.0448837	0.060103	0.0370192
94006_at	Azi2	0.0451218	0.0946929	0.0402671
103727_at	Hrb	0.0451641	0.060103	0.0390001
93704_at	Foxf1a	0.0453956	0.060103	0.0499382
97380_at	1700016A15Rik	0.0454675	0.0367141	0.061161
92383_at	Dyrk1a	0.0455833	0.060103	0.0432495
160210_at	4930539H15Rik	0.0456036	0.060103	0.0484802
100733_at	Psma2	0.0458152	0.0367141	0.0268913
97394_at	Smarca5	0.0458626	0.0121856	0.012707
95052_at	1110035L05Rik	0.0459394	0.0367141	0.0354185
99645_at	4921506J03Rik	0.046355	0.0367141	0.0340301
92640_at	Vps35	0.0465367	0.0946929	0.0617208
93859_at	Mtif2	0.0465668	0.210075	0.035869
104311_at	Ubxd2	0.046608	0.060103	0.0522154
95387_f_at	Sema4b	0.0471546	0.0367141	0.0307721
93841_at	D3Ertd194e	0.0473294	0.0367141	0.0390211
93308_s_at	Pcx	0.0475135	0.060103	0.097915
103862_r_at	D7Wsu128e	0.0475674	0.060103	0.04707
97857_at	Zdhhc3	0.0478935	0.0367141	0.0382738
92534_at	Gem	0.048225	0.060103	0.0346911
98055_at	Blcap	0.0482517	0.0367141	0.0856926
101631_at	Sox11	0.0486131	0.0121856	0.0188436
96023_at	D8Wsu151e	0.0490165	0.060103	0.0532127
93325_at	Polr2e	0.0494689	0.060103	0.0378598
96545_s_at	A730042J05Rik	0.0495827	0.060103	0.0997466
160966_at	---	0.0495877	0.060103	0.0358235
95110_at	Ppil2	0.0498469	0.0367141	0.0321055

TableS4. Mann-Whitney p values for genes dysregulated in term T1α (-/-) lungs compared to wildtype with Student's t test p less 0.05.

affy id	Gene Symbol	t-test	mann-whitney	log t-test
92777_at	Cyr61	0.00091941	0.0808557	0.0161513
102952_g_at	Cradd	0.00630042	0.0808557	0.00807013
98530_at	Gas5	0.00684195	0.0808557	0.014112
99571_at	MGC29978	0.00788286	0.0808557	0.00435574
161119_at	Epha5	0.00921131	0.0808557	0.0157583
92597_s_at	Atp6v1b2	0.0113043	0.0808557	0.0220283
103762_at	2810405L04Rik	0.0114954	0.0808557	0.022912
98579_at	Egr1	0.0125173	0.0808557	0.0594682
104586_at	5730507C05Rik	0.0129271	0.0808557	0.00992588
97836_at	Rnf7	0.01295	0.0808557	0.0239484
95358_at	Pip5k2a	0.0174109	0.0808557	0.0125388
99435_at	Fgf7	0.0179117	0.0808557	0.026702
94347_i_at	Pcmt1	0.0194135	0.0808557	0.0259424
97515_at	Hsd17b4	0.0203854	0.0808557	0.0181164
94499_at	Mgea5	0.0207296	0.0808557	0.0311768
160657_at	Ilf3	0.0218611	0.0808557	0.0144424
99528_at	Spin	0.0226791	0.0808557	0.0348026
94110_f_at	D10627	0.0229913	0.0808557	0.0141277
103990_at	Fosb	0.0234498	0.0808557	0.0368113
162387_f_at	Mfn1	0.0238109	0.0808557	0.0370792
95101_at	Tde2	0.0246686	0.0808557	0.0274475
100629_at	Gstm5	0.0255361	0.0808557	0.02906
94356_at	Trp53bp1	0.0277974	0.0808557	0.025794
103052_r_at	Nr2f2	0.0279743	0.0808557	0.0463541
99909_at	Trpc6	0.0295727	0.0808557	0.0698773
104317_at	Cse1l	0.0319415	0.0808557	0.0388737
104246_at	Asxl1	0.0322854	0.0808557	0.0157165
100911_at	Gpaa1	0.0333297	0.0808557	0.0319338
100886_f_at	Mrpl45	0.0335638	0.0808557	0.0436676
104059_at	5830451P18Rik	0.0340383	0.0808557	0.0266965
99929_at	Ext2	0.0345782	0.0808557	0.0268833
103225_at	2310005K03Rik	0.0349462	0.0808557	0.0482057
103887_at	S100a9	0.0352857	0.0808557	0.0246118
101930_at	Nfix	0.0357645	0.0808557	0.0281825
100929_at	---	0.0384704	0.0808557	0.0253617
95360_at	Zcchc6	0.0390437	0.0808557	0.0251623
94448_at	Bcl10	0.0407314	0.0808557	0.0464922
104598_at	Dusp1	0.0423292	0.0808557	0.0297359
94844_at	Rpl39	0.0423751	0.0808557	0.0582192
161638_f_at	Prkcabp	0.0429222	0.0808557	0.041656
94034_at	Smfn	0.0430504	0.0808557	0.0336115
160324_at	Rpa3	0.0444131	0.0808557	0.0618169
161858_f_at	Rb1	0.044704	0.0808557	0.0424872
102626_r_at	Zfp54	0.0463506	0.0808557	0.0371274
97776_at	Drd2	0.0473252	0.0808557	0.0603478
160717_at	1110059E24Rik	0.0473456	0.0808557	0.0587767
100004_at	5930412E23Rik	0.0473461	0.0808557	0.0574428
94224_s_at	Ifi205	0.0483283	0.0808557	0.110278

98112_r_at	Lap3	0.0490379	0.0808557	0.0569943
102371_at	Nr4a1	0.0498874	0.0808557	0.139428
