# Supplementary material for: Alterations in gene expression in T1α null lung: a model of deficient alveolar sac development
Source: BMC Dev Biol. 2006 Jul 25;6:35. doi: 10.1186/1471-213X-6-35 (PMC1562362; doi:10.1186/1471-213X-6-35)
Supplement: Additional File 1 — Figure S1. Ratios of epithelial vs. mesenchymal marker genes in T1α (+/+) and (-/-) lungs at E18.5 and term. [file 1471-213X-6-35-S1.pdf]

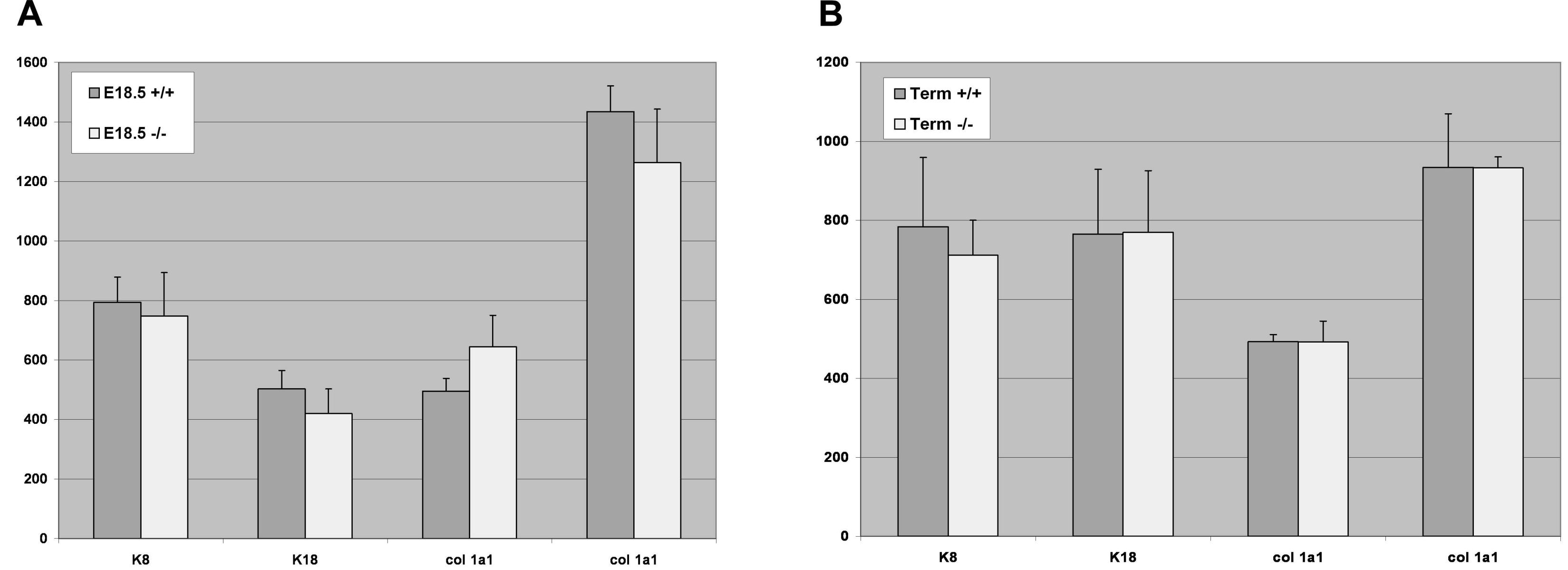


**Figure S1.** Microarray data for the epithelial genes cytokeratin 8 (K8), cytokeratin 18 (K18) and the mesenchymal gene collagen 1$\alpha$1 (two probe sets) in T1$\alpha$ (+/+, blue) and (-/-, light blue) lungs at (A) E18.5 and (B) term.
