# Supplementary material for: Alterations in gene expression in T1α null lung: a model of deficient alveolar sac development
Source: BMC Dev Biol. 2006 Jul 25;6:35. doi: 10.1186/1471-213X-6-35 (PMC1562362; doi:10.1186/1471-213X-6-35)
Supplement: Additional File 2 — References in Table 1 and Table 2. The references include lung expression of specific genes. [file 1471-213X-6-35-S2.pdf]

References in Table1 and Table2

E1. Cao YX, Ramirez MI, Williams MC. Enhanced binding of sp1/sp3 transcription factors mediates the hyperoxia-induced increased expression of the lung type i cell gene t1alpha. *J Cell Biochem* 2003;89(5):887-901.

E2. Sock E, Rettig SD, Enderich J, Bosl MR, Tamm ER, Wegner M. Gene targeting reveals a widespread role for the high-mobility-group transcription factor sox11 in tissue remodeling. *Mol Cell Biol* 2004;24(15):6635-6644.

E3. Chetty A, Andersson S, Lassus P, Nielsen HC. Insulin-like growth factor-1 (igf-1) and igf-1 receptor (igf-1r) expression in human lung in rds and bpd. *Pediatr Pulmonol* 2004;37(2):128-136.

E4. Powell-Braxton L, Hollingshead P, Warburton C, Dowd M, Pitts-Meek S, Dalton D, Gillett N, Stewart TA. Igf-i is required for normal embryonic growth in mice. *Genes Dev* 1993;7(12B):2609-2617.

E5. Grizzi F, Chiriva-Internati M, Franceschini B, Bumm K, Colombo P, Ciccarelli M, Donetti E, Gagliano N, Hermonat PL, Bright RK, et al. Sperm protein 17 is expressed in human somatic ciliated epithelia. *J Histochem Cytochem* 2004;52(4):549-554.

E6. Wen Y, Richardson RT, Widgren EE, O'Rand MG. Characterization of sp17: A ubiquitous three domain protein that binds heparin. *Biochem J* 2001;357(Pt 1):25-31.

E7. Olson DM, Sheth MV, Rodrigo MC, Burghardt JS, Eyster KM. Effect of developmental age and hyperoxia exposure on kinase and phosphatase activities in newborn rat lungs. *Exp Lung Res* 1998;24(3):339-353.

E9. Lu J, Qian J, Izvolsky KI, Cardoso WV. Global analysis of genes differentially expressed in branching and non-branching regions of the mouse embryonic lung. *Dev Biol* 2004;273(2):418-435.

E9. Yoshida T, Takanari H, Izutsu K. Distribution of cytoplasmic and axonemal dyneins in rat tissues. *J Cell Sci* 1992;101 (Pt 3):579-587.

E10. Wickman K, Seldin MF, James MR, Gendler SJ, Clapham DE. Partial structure, chromosome localization, and expression of the mouse icln gene. *Genomics* 1997;40(3):402-408.

E11. Wang P, Wu P, Egan RW, Billah MM. Cloning, characterization, and tissue distribution of mouse phosphodiesterase 7a1. *Biochem Biophys Res Commun* 2000;276(3):1271-1277.

E12. Lama V, Moore BB, Christensen P, Toews GB, Peters-Golden M. Prostaglandin e2 synthesis and suppression of fibroblast proliferation by alveolar epithelial cells is cyclooxygenase-2-dependent. *Am J Respir Cell Mol Biol* 2002;27(6):752-758.

E13. Ono R, Shiura H, Aburatani H, Kohda T, Kaneko-Ishino T, Ishino F. Identification of a large novel imprinted gene cluster on mouse proximal chromosome 6. *Genome Res* 2003;13(7):1696-1705.

E14. Chen ZL, Momota Y, Kato K, Taniguchi M, Inoue N, Shiosaka S, Yoshida S. Expression of neuropsin mrna in the mouse embryo and the pregnant uterus. *J Histochem Cytochem* 1998;46(3):313-320.
E15. Schisler NJ, Singh SM. Inheritance and expression of tissue-specific catalase activity during development and aging in mice. *Genome* 1987;29(5):748-760.
E16. Warner DR, Mozier NM, Pearson JD, Hoffman JL. Cloning and base sequence analysis of a cdna encoding mouse lung thioether s-methyltransferase. *Biochim Biophys Acta* 1995;1246(2):160-166.
E17. Xu J, Burgoyne PS, Arnold AP. Sex differences in sex chromosome gene expression in mouse brain. *Hum Mol Genet* 2002;11(12):1409-1419.
E18. Nakamura T, Yabe D, Kanazawa N, Tashiro K, Sasayama S, Honjo T. Molecular cloning, characterization, and chromosomal localization of fkbp23, a novel fk506-binding protein with ca2+-binding ability. *Genomics* 1998;54(1):89-98.
E19. Kwee L, Baldwin HS, Shen HM, Stewart CL, Buck C, Buck CA, Labow MA. Defective development of the embryonic and extraembryonic circulatory systems in vascular cell adhesion molecule (vcam-1) deficient mice. *Development* 1995;121(2):489-503.
E20. Kalinichenko VV, Gusarova GA, Kim IM, Shin B, Yoder HM, Clark J, Sapozhnikov AM, Whitsett JA, Costa RH. Foxf1 haploinsufficiency reduces notch-2 signaling during mouse lung development. *Am J Physiol Lung Cell Mol Physiol* 2004;286(3):L521-530.
E21. Cerretti DP, Vanden Bos T, Nelson N, Kozlosky CJ, Reddy P, Maraskovsky E, Park LS, Lyman SD, Copeland NG, Gilbert DJ, et al. Isolation of lerk-5: A ligand of the eph-related receptor tyrosine kinases. *Mol Immunol* 1995;32(16):1197-1205.
E22. Lazzaro MA, Picketts DJ. Cloning and characterization of the murine imitation switch (iswi) genes: Differential expression patterns suggest distinct developmental roles for snf2h and snf2l. *J Neurochem* 2001;77(4):1145-1156.
E23. Rodriguez-Manzaneque JC, Perez-Castillo A, Santos A. Control by thyroid hormone of ngfi-a gene expression in lung: Regulation of ngfi-a promoter activity. *Mol Cell Endocrinol* 1998;141(1-2):101-110.
E24. Lee CG, Cho SJ, Kang MJ, Chapoval SP, Lee PJ, Noble PW, Yehualaeshet T, Lu B, Flavell RA, Milbrandt J, et al. Early growth response gene 1-mediated apoptosis is essential for transforming growth factor beta1-induced pulmonary fibrosis. *J Exp Med* 2004;200(3):377-389.
E25. Shin HJ, Lee BH, Yeo MG, Oh SH, Park JD, Park KK, Chung JH, Moon CK, Lee MO. Induction of orphan nuclear receptor nur77 gene expression and its role in cadmium-induced apoptosis in lung. *Carcinogenesis* 2004;25(8):1467-1475.
E26. Wu Q, Li Y, Liu R, Agadir A, Lee MO, Liu Y, Zhang X. Modulation of retinoic acid sensitivity in lung cancer cells through dynamic balance of orphan receptors nur77 and coup-tf and their heterodimerization. *Embo J* 1997;16(7):1656-1669.

E27. Reddy SP, Mossman BT. Role and regulation of activator protein-1 in toxicant-induced responses of the lung. *Am J Physiol Lung Cell Mol Physiol* 2002;283(6):L1161-1178.
E28. Levine RA, Hopman T, Guo L, Chang MJ, Johnson N. Induction of retinoblastoma gene expression during terminal growth arrest of a conditionally immortalized fetal rat lung epithelial cell line and during fetal lung maturation. *Exp Cell Res* 1998;239(2):264-276.
E29. Li M, Zhou JY, Ge Y, Matherly LH, Wu GS. The phosphatase mkp1 is a transcriptional target of p53 involved in cell cycle regulation. *J Biol Chem* 2003;278(42):41059-41068.
E30. Kireeva ML, Latinkic BV, Kolesnikova TV, Chen CC, Yang GP, Abler AS, Lau LF. Cyr61 and fisp12 are both ecm-associated signaling molecules: Activities, metabolism, and localization during development. *Exp Cell Res* 1997;233(1):63-77.
E31. Fehrenbach H, Kasper M, Tschernig T, Pan T, Schuh D, Shannon JM, Muller M, Mason RJ. Keratinocyte growth factor-induced hyperplasia of rat alveolar type ii cells in vivo is resolved by differentiation into type i cells and by apoptosis. *Eur Respir J* 1999;14(3):534-544.
E32. Ware LB, Matthay MA. Keratinocyte and hepatocyte growth factors in the lung: Roles in lung development, inflammation, and repair. *Am J Physiol Lung Cell Mol Physiol* 2002;282(5):L924-940.
E33. Freichel M, Vennekens R, Olausson J, Hoffmann M, Muller C, Stolz S, Scheunemann J, Weissgerber P, Flockerzi V. Functional role of trpc proteins in vivo: Lessons from trpc-deficient mouse models. *Biochem Biophys Res Commun* 2004;322(4):1352-1358.
E34. Puopolo K, Kumamoto C, Adachi I, Magner R, Forgac M. Differential expression of the "b" subunit of the vacuolar h(+)-atpase in bovine tissues. *J Biol Chem* 1992;267(6):3696-3706.
E35. Nolte C, Moos M, Schachner M. Immunolocalization of the neural cell adhesion molecule l1 in epithelia of rodents. *Cell Tissue Res* 1999;298(2):261-273.
36. Coccia EM, Cicala C, Charlesworth A, Ciccarelli C, Rossi GB, Philipson L, Sorrentino V. Regulation and expression of a growth arrest-specific gene (gas5) during growth, differentiation, and development. *Mol Cell Biol* 1992;12(8):3514-3521.
